# Driftage: a multi-agent system framework for concept drift detection

**DOI:** 10.1093/gigascience/giab030

**Published:** 2021-06-01

**Authors:** Diogo Munaro Vieira, Chrystinne Fernandes, Carlos Lucena, Sérgio Lifschitz

**Affiliations:** Informatics Department, Pontifical Catholic University of Rio de Janeiro (PUC-Rio), Marques de São Vicente, 225, Gávea, Rio de Janeiro, RJ 22451-900, Brazil; Informatics Department, Pontifical Catholic University of Rio de Janeiro (PUC-Rio), Marques de São Vicente, 225, Gávea, Rio de Janeiro, RJ 22451-900, Brazil; Informatics Department, Pontifical Catholic University of Rio de Janeiro (PUC-Rio), Marques de São Vicente, 225, Gávea, Rio de Janeiro, RJ 22451-900, Brazil; Informatics Department, Pontifical Catholic University of Rio de Janeiro (PUC-Rio), Marques de São Vicente, 225, Gávea, Rio de Janeiro, RJ 22451-900, Brazil

**Keywords:** concept drift, data drift, anomaly detection, time series, multi-agent systems, data mining, machine learning interpretability, machine learning explainability

## Abstract

**Background:**

The amount of data and behavior changes in society happens at a swift pace in this interconnected world. Consequently, machine learning algorithms lose accuracy because they do not know these new patterns. This change in the data pattern is known as concept drift. There exist many approaches for dealing with these drifts. Usually, these methods are costly to implement because they require (i) knowledge of drift detection algorithms, (ii) software engineering strategies, and (iii) continuous maintenance concerning new drifts.

**Results:**

This article proposes to create Driftage: a new framework using multi-agent systems to simplify the implementation of concept drift detectors considerably and divide concept drift detection responsibilities between agents, enhancing explainability of each part of drift detection. As a case study, we illustrate our strategy using a muscle activity monitor of electromyography. We show a reduction in the number of false-positive drifts detected, improving detection interpretability, and enabling concept drift detectors’ interactivity with other knowledge bases.

**Conclusion:**

We conclude that using Driftage, arises a new paradigm to implement concept drift algorithms with multi-agent architecture that contributes to split drift detection responsability, algorithms interpretability and more dynamic algorithms adaptation.

## Introduction

In muscular monitoring activity, electromyography (EMG) has long been the primary technique to measure the action potential from muscle cells [1]. Today, several sports use EMG in such contexts as monitoring soccer players' athletic activity or seeking better performance in racehorses [2,3].

Machine learning techniques have been applied to EMG time-series data because health monitoring needs fast insights because the patient could need emergency assistance [4, 5]. These data have a lot of spikes, and patterns are complicated to understand. Time-series data with continuous tips are also hard to learn because all the peaks are similar to outliers or anomalies. Still, these peaks happen all the time, and we claim that algorithms need to be able to distinguish them. For this purpose, concept drift strategies appear to analyse automatically streaming time-series data [6–8].

There are many types of drifts in the concept drift detection (CDD) area [7, 9, 10]. Within EMG, sudden, gradual, recurring, or incremental drifts can be detected because a potential muscular activity is very reactive. When you make a move, many muscles react to it [11, 12]. There are many ways to detect each type of these drifts, and it is not elementary to build an algorithm that will detect everything. In concept drifts, researchers have developed supervised [13, 14], semi-supervised [15], unsupervised [16, 17, 18], statistical [19, 20], or even evolutionary algorithms [21] to deal with these drifts, but none of them is perfect for all drift types.

Some publications are arising with machine learning ensembles for CDD because of the nature of the data that these detectors need to adapt to [22–25]. There are several factors such as data seasonality or change of data drift type; these ensembles can choose the best estimator for each case, and each estimator can still act alone. Nevertheless, this approach necessitates retraining of base learners and strategies to select the best estimator that can affect detection speed [22, 26].

One approach to designing adaptive software is using the MAPE-K (Monitor-Analyse-Plan-Execute over a shared Knowledge) software pattern for self-aware systems [27–30]. MAPE-K is organized into 4 components:

- The "Monitor" is responsible for environmental monitoring, basically capturing data from sensors or what else the software knows about the environment and stores on the knowledge base (KB);
- The "Analyser" will enrich knowledge using the collected data from the environment and reporting to the KB the result of its analysis;
- The "Planner" understands the analysis made by analysers and makes decisions on it while saving this information into the KB; and
- The "Executor" gets decisions from the KB and knows how to execute them. The most common representation for the executor is an actuator.

The KB is unnecessary for all components on MAPE-K, but all systems need to communicate and share information [31]. MAPE-K software architecture was recently used to model an agent on multi-agent systems (MAS) [32]. However, in this work, each agent is a component of MAPE-K architecture.

Traditionally, a MAS comprises agents, each of which autonomously learns from the environment and exchanges messages with others. This structure is great for solving complex problems [33, 34]. There are some obstacles in the MAS field because of the complexity imposed when multiple smart agents communicate, particularly in distributed environments. Some of these challenges test the system [35] and flow consistency [36]. Moreover, streaming architectures using MAS that guide this work with best practices add less responsibility for each agent, splitting intelligence between more specific agents [37, 38].

There is a lot of synergy with CDD and MAS ensembles because each agent can communicate with others and analyse the data individually to determine whether the drift was detected. Some studies using MAS with ensemble strategies have already been performed [39, 40], but none are focused on CDD. Also, there are multiple architectures for a MAS that can be chosen to elaborate an agent-oriented software. The major challenge involves finding a good one that learns with the environment and solves the problem [33, 41, 42].

The more the architecture for these systems is enhanced, the more complicated the system gets. In addition, there is a greater chance of losing control, resulting in mistakes in the production environment, even with some methods already described to avoid this [43, 44]. Detecting concept drifts on data streams in a scalable model for production environments is time consuming because you need to build CDD algorithms and be aware of the data pipeline, data ingestion, and drift detection results [45].

To solve the dependency of data engineering pipelines and customized CDD machine learning algorithms, we propose here Driftage, a modular multi-agent framework for CDD with only certain types of agents that can be implemented with specialized functions deriving in other agents. Focus on a process is the best practice for MAS implementation to avoid the complexity of multiple agents and improve software reuse [46, 47].

It is very complex to explain what is happening inside machine learning ensemble algorithms and interpret their results because they consist of a mixture of multiple machine learning techniques and algorithms [48]. In this article, we propose a method to modularize these ensembles into various MAS agents with a much more straightforward way to explain the results of each agent's model, as well as the ensemble algorithm. This MAS modularity provides both explainability for each agent’s machine learning model and interpretability for its effects. Explainability and interpretability for each agent enable us to do the same for the whole system.

A case study with Driftage was created in this article with EMG data to validate how MAS software architecture helps detect concept drifts on muscular activity during a punching exercise, enabling split efforts between scalability and drift detection issues during CDD challenges with a highly extensible and modular framework.

## Methods

### Driftage architecture

Driftage is a modular framework based on MAPE-K, chosen as the pattern to model this agent-based framework because CDD needs high adaptability and fits very well with MAS.

Each agent type in Driftage has only 1 accountable agent on the MAPE-K architecture. Each agent can be implemented to follow the selected goal without affecting the others but can exchange information with others. Instead of an agent using the MAPE-K software pattern, an agent on the Driftage framework can be implemented following 1 of the 4 types: Monitor, Analyser, Planner, or Executor. Each type can generate multiple autonomous agents.

There are 2 main flows on this framework:

- Monitor–Analyser: for capture and fast prediction of concept drifts on data;
- Planner–Executor: to analyse whether concept drift detected should be alerted.

These 2 flows can intercommunicate by means of a KB, where drifts are stored, and we make all history about drift analysis persistent. Each agent communicates through an XMPP server on the framework because the implementation extends Spade [49], which is a library for MAS using Python. The XMPP protocol solves some problems with MAS, already providing authentication and communication channels for the agents. XMPP servers also work for load balancing and guarantee message exchanges.

**Figure 1:**
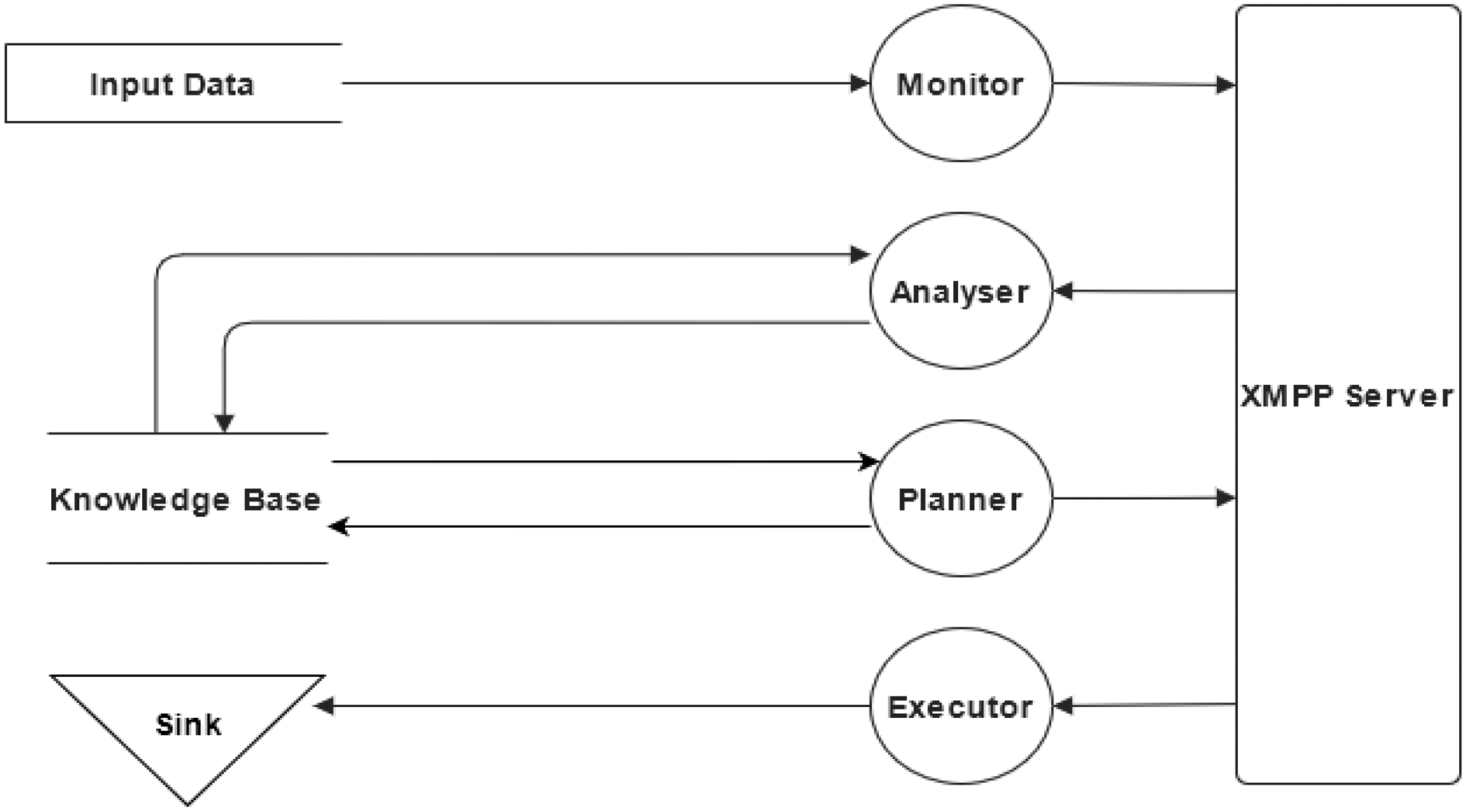
Driftage data flow for concept drift detection. All agents communicate through XMPP server, and only the Analyser and the Planner can use information from the Knowledge Base.

We have implemented Driftage using Python because data engineers widely use it, and it enables the programmer to answer the system’s requirements. The data flow for this framework is described in Fig. 1. The next section describes how we structure the KB for data sharing between flows Monitor–Analyser and Planner–Executor. These 2 agent communications flows are further explained in the following subsections of the Methods.

#### Knowledge Base

On the MAPE-K pattern, systems use shared knowledge that is implemented on the Driftage architecture by a database. This database stores all concept drifts that are detected and whom they were detected by, with the schema shown in Table 1.

**Table 1.** Schema of data saved in Knowledge Base

Column	Description	Example
jid	Name of the Analyser that predicts data as drift for each collected piece of data	Custom drift analyser
data	Data collected and sent to Analyser by Monitor in JSON format	{”sensor”:429}
datetime_monitored	When the data were collected by Monitor	2020-07-21 14:36:00
datetime_analysed	When the data were analysed by Analyser	2020-07-21 14:37:00
identifier	Identifier from data collected that identifies which data are monitored	left_thigh
predicted	Boolean prediction of data by Analyser representing whether a concept drift was detected	False

This schema works for any relational database, and even for non-relational, which SQLAlchemy [50] supports.

Stored data collected and predicted by Analyser can be queried for retraining or by the Planner to improve the researcher's predictions. This way, we can connect the 2 flows. Only the Analyser and Planner know how to connect to KB.

#### Monitor–Analyser

This first flow is responsible for capturing data and detecting the concept drift. After drift detection, this flow saves the result on KB.

Monitor agents capture the data integrated into any desired framework: Spark, Flink, or even a Python function. Our framework sends these collected data to every Analyser that asks for it. The Analyser subscribes to Monitors to receive the data collected and analyses them using a customized predictor for CDD. Fast classifiers from Scikit-Multiflow or Facebook Prophet can be attached as a predictor.

**Figure 2:**
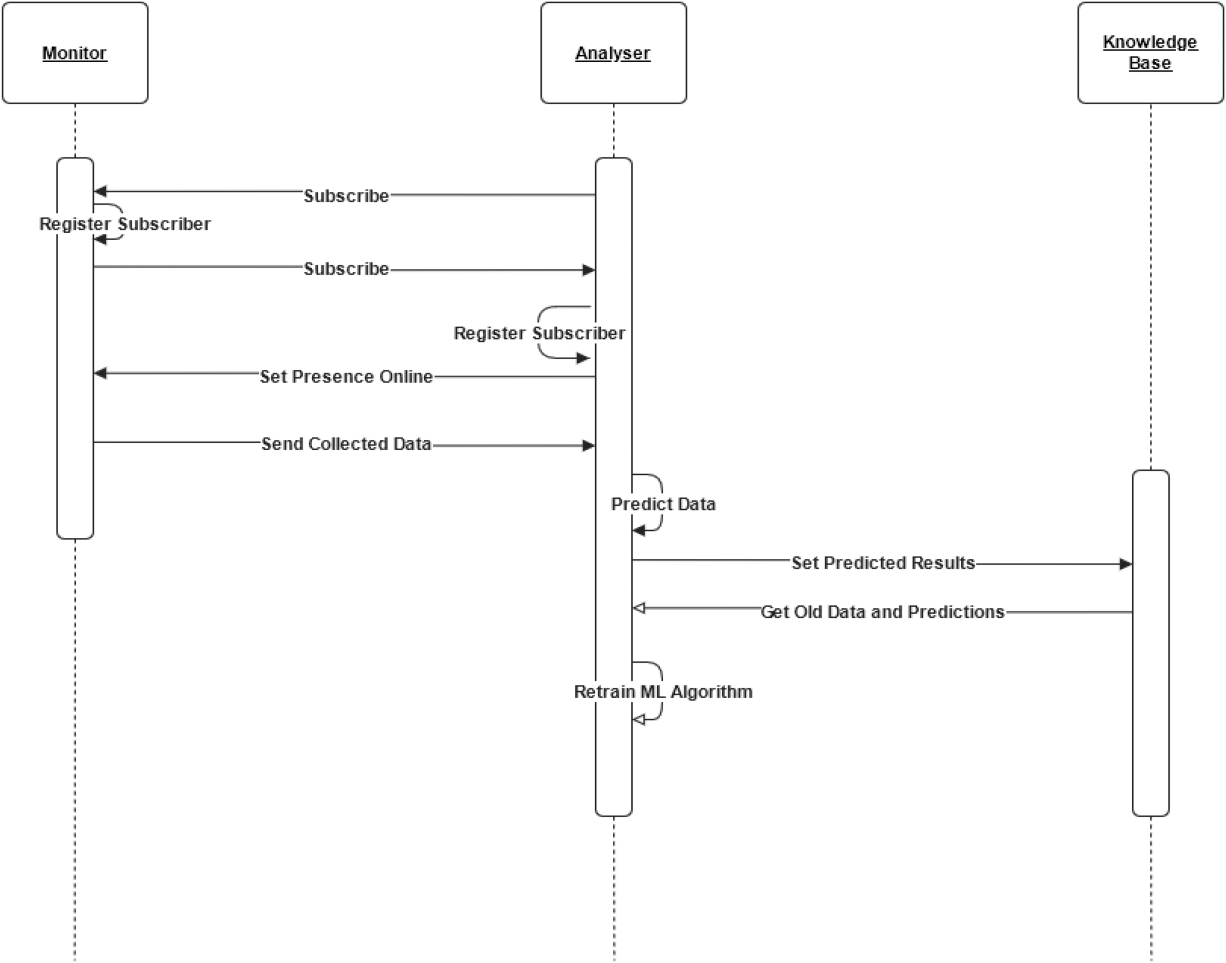
Monitor–Analyser flow. The Monitor agent collects data and sends them to the Analyser agent only if the Analyser is available, then the Analyser makes predictions and saves on KB. The Analyser agent can consult KB for retraining of the researcher's predictor. ML: machine learning.

The flow is shown as a sequence diagram in Fig. 2. After Analyser agents have subscribed to Monitors, Monitors subscribe to Analysers too because they need to know whether Analysers are working to send new data. When Analysers receive data from Monitors, they can predict those data and store them in the KB. There is another asynchronous task for the Analyser algorithm retraining that happens systematically.

#### Planner–Executor

**Figure 3:**
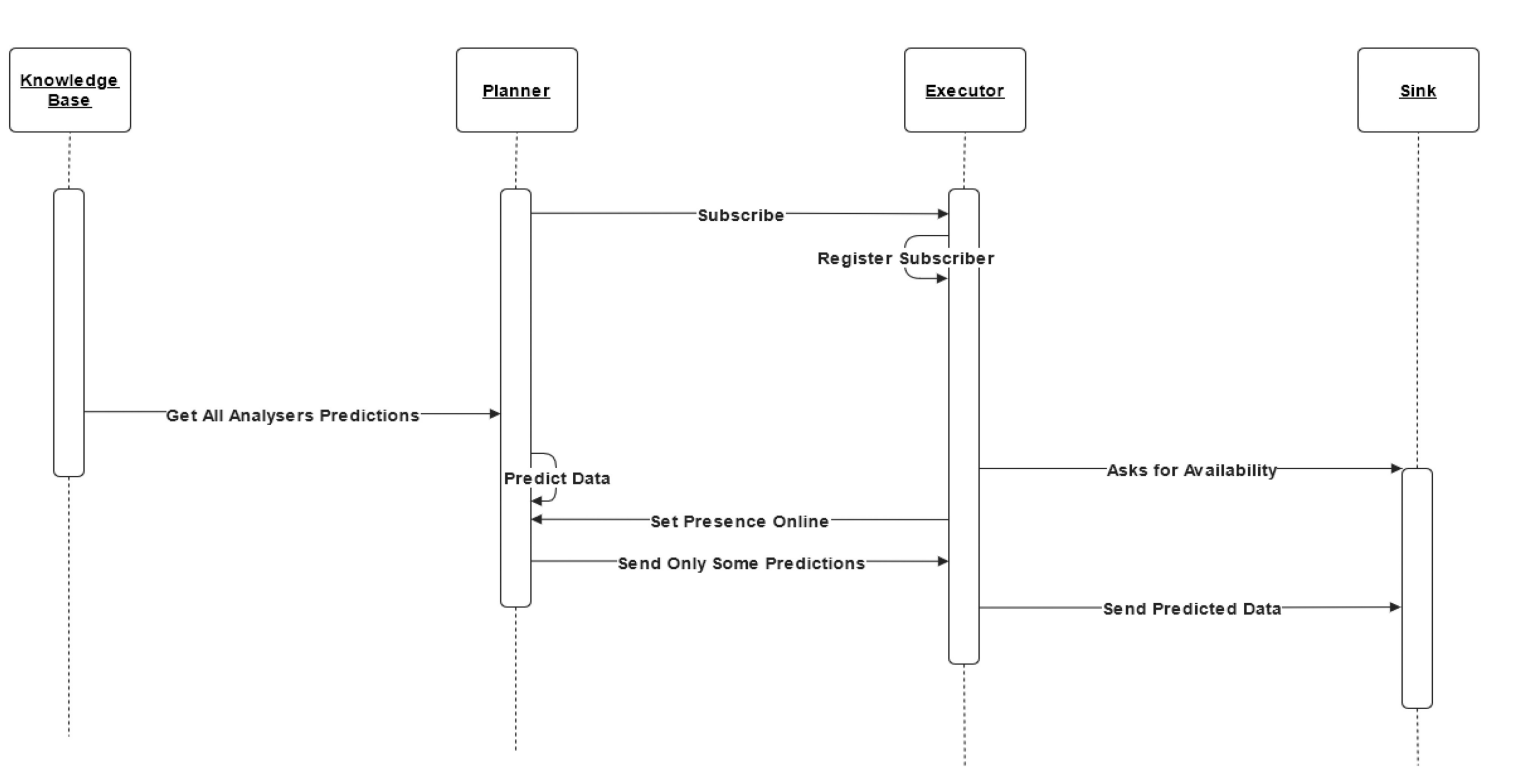
Planner–Executor flow. The Planner agent communicates with KB to get new predictions while the Executor agent asks Planner for concept drift detection results only if Sink is alive.

This last flow is responsible for alerting about drifts detected. It queries KB and decides whether a drift detection should be informed. The Planner agents keep observing for new predictions and, based on them, decides whether the drift is valid. If it is an actual drift, it should be sent to the Executor. A custom predictor can be created for this purpose too, like a voting one or a more time-consuming algorithm from Scikit-Learn, TensorFlow, PyTorch, etc. Executor agents are subscribed from Planners and receive from them new drifts to send to a custom Sink. This Sink can be an Apache Kafka, RabbitMQ, API, and so forth. The Executor knows whether a Sink is available and informs the Planner regarding whether it can handle new drifts. We consider a sequence diagram to describe this flow in Fig. 3.

#### Analyser and Planner interaction

**Figure 4:**
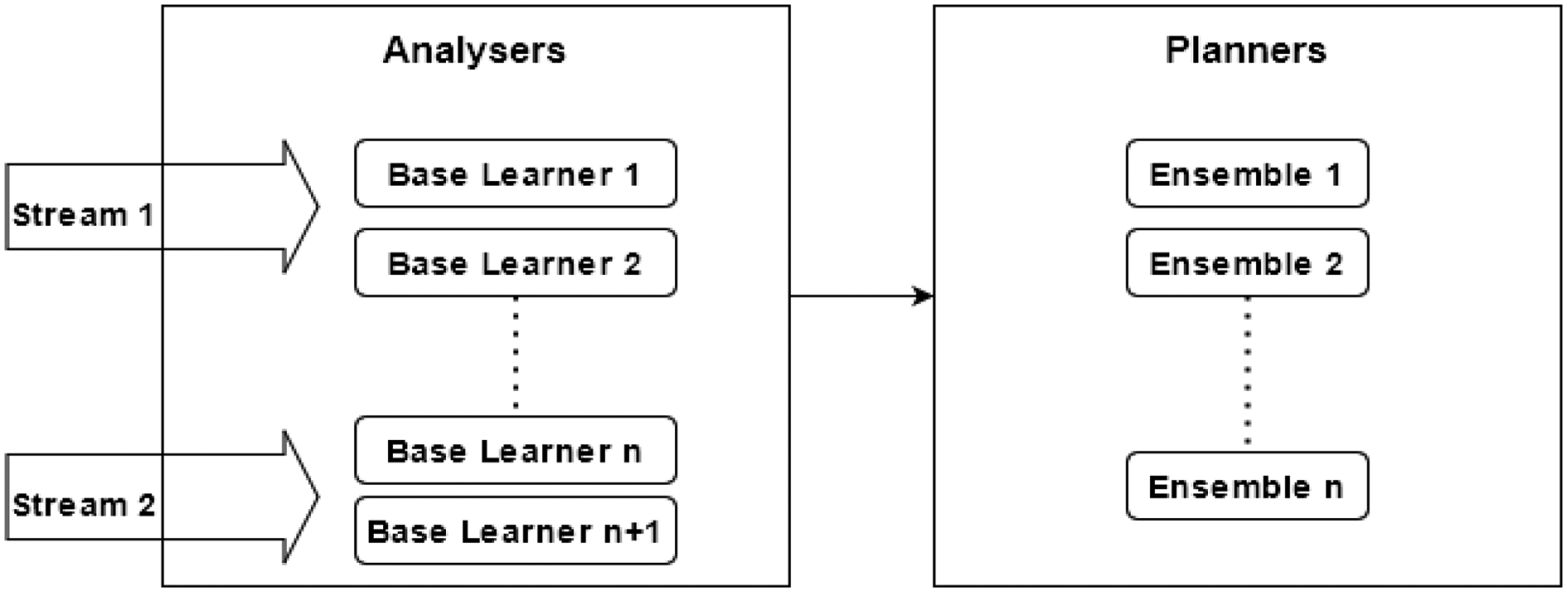
Driftage multi-agent ensemble abstraction and each stream being handled by 2 base learners.

MAS distributed architecture makes possible a lot of combinations on stream evaluation. Figure 4 presents an example of a combination that uses the Analysers as base learners classifying CDD on streams for further evaluation by Planner with an ensemble algorithm. Each Planner combines results from multiple Analysers that are evaluating 1 stream and can change the Analyser subscription, ignoring its evaluation or adding new Analysers depending on ensemble algorithm. Multiple Planners can watch the same Analysers using different Executors to save information about concept drift. This function is useful when it is necessary to configure multiple algorithms for CDD with different sensibilities. Some machine learning models are more sensitive to data drifts than others that can wait for more aggressive data changes.

**Figure 5:**
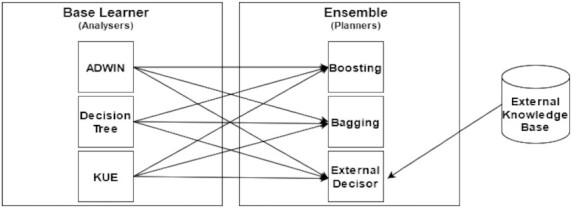
Every base learner and ensemble method is registered alone, and the result can be interpretable without the influence of other algorithms.

The Driftage framework gives CDD models flexibility and interpretability because each part of the CDD evaluation model can be evaluated and metrified alone. This is useful for debugging models and elucidating what is happening on the evaluation pipeline. For example, ensemble algorithms such as Kappa Updated Ensemble (KUE) or Adaptive Random Forest [24, 25] can be implemented as Analysers combined by another ensemble algorithms on Planner. This Planner can be an ensemble algorithm or just get outside information from another KB as illustrated in Fig. 5.

### Data acquisition

There is an open dataset for EMG on UCI Machine Learning Repository [51] with information on the activity of 8 muscles during various exercises such as running, punching, or jumping. For this article, we have chosen the punching activity file with 9.637 instances, where each instance was considered as a microsecond action potential of a muscle cell. This action potential is registered in microvolts at each line of the CSV file.

## Health Monitor Results

This section shows the architecture proposed for a health monitor of muscle cells, followed by the implementation of the CDD algorithm and the results of this algorithm on the UCI dataset to validate the Driftage conception and architecture.

### Architecture design

Health monitoring is a complicated task, and it is hard to know the best time to send an alert about patient health changes because of the challenges involved in collecting the data, analysing them for drifts, finding the best way to communicate the drift, and really sending the communication. There needs to be >1 alteration in a muscular activity before it can indicate a muscular disease or even a difference in a patient’s movement. For example, a person doing exercises may slightly alter the direction of movement, but it is not characterized as muscular fatigue. An Analyser specialized for each muscle activity was developed to understand each athletic activity data input and a Planner that detects whether the Executor should send a concept drift alert.

The MAPE-K–based MAS framework proposed here makes it simple to split each responsibility from the CDD. Each agent on the architecture is responsible for a particular task: collecting the data (Monitor), analysing the drift on the data (Analyser), deciding what to do with the detected drifts (Planner), and esending an alert when called for (Executor). Each agent can use relevant Python tools to implement the best solution for each case.

Many frameworks and tools already solve monitoring and capturing data, so integrating with Apache Spark is the most straightforward approach for Python projects using PySpark. Monitors were implemented by combining with PySpark using 1 Monitor for each kind of muscle. For each row in a CSV file, there are 8 sensor signals for each type of muscle, and Spark executors send sensor data for the corresponding Monitor. Each Monitor sends data to 1 Analyser but could send to others if needed. In this project design, each Analyser covers 1 muscle, analysing concept drifts on it.

As long as each Analyser knows only about an individual's muscle activity, the Planner is simple and predicts a concept drift if ≥2 muscles have a drift detected. We chose 2 muscles because the dataset has 4 muscles for arms and legs, 2 for each side (left and right), so if the patient has a problem on 1 side of the body, ≥2 muscles should be affected. To avoid some “cold start” problems, the Planner also ignores the situation in which all the muscles indicate a drift, so the rule is 2 ≥ *n*_drift_ < 8, where *n*_drift_ is the number of muscles with drift detected. Finally, the Executor was built saving drifts on a file as CSV and validating if the file system is available to write data.

**Figure 6:**
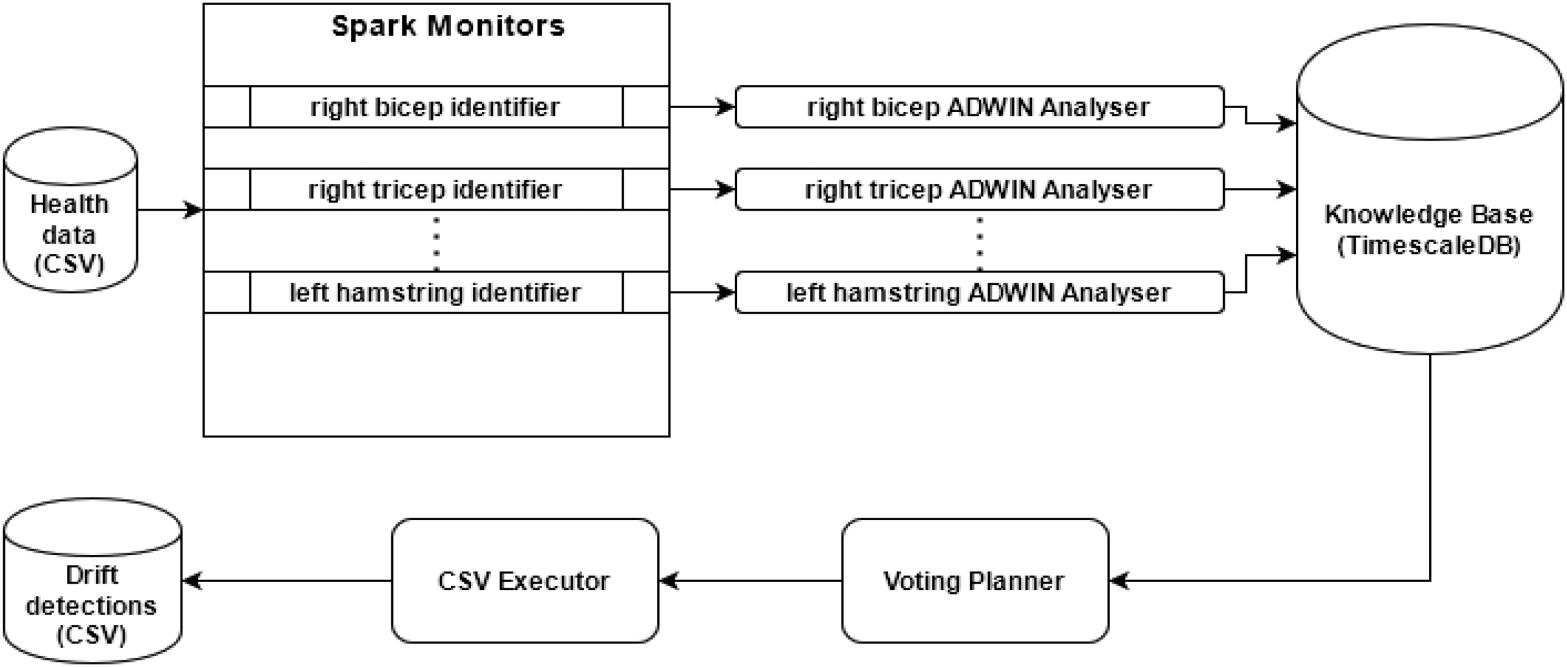
Health Monitor implementation. CSV file is ingested by Monitors, then concept drifts are predicted by Analysers, then saved on KB. Planners get predictions and vote on whether it is a concept drift, then the Executors save detected drifts on another CSV file.

Docker containers are used to support each agent, so each Monitor, Analyser, Planner, and Executor implementation uses Docker to enhance reproducibility and provide more effortless scalability. TimescaleDB as KB also used Docker containers during this experiment, but we recommend storing data in a better way on production environments. All this architecture design is illustrated in Fig. 6.

### Drift detection algorithm

One of the most famous CDD algorithms is ADWIN (adaptive sliding window algorithm) [52]. It efficiently keeps a variable-length window of recent items, whose contents can be compared to discern whether there has been any change in the data distribution. This window is further divided into 2 subwindows (*W*_0_, *W*_1_) used to determine whether a change has happened. ADWIN compares the average of *W*_0_ and *W*_1_ to confirm that they correspond to the same distribution. Concept drift is detected if the distribution equality no longer holds. Upon detecting a drift, *W*_0_ is replaced by *W*_1_ and a new *W*_1_ is initialized. ADWIN uses a confidence value δ ∈ (0, 1) to determine whether the 2 subwindows correspond to the same distribution and δ is a stateless parameter in the algorithm that just contributes for CDD in the moment of comparing the subwindows. ADWIN will be our base learner on ensemble architecture.

**Algorithm 1:**
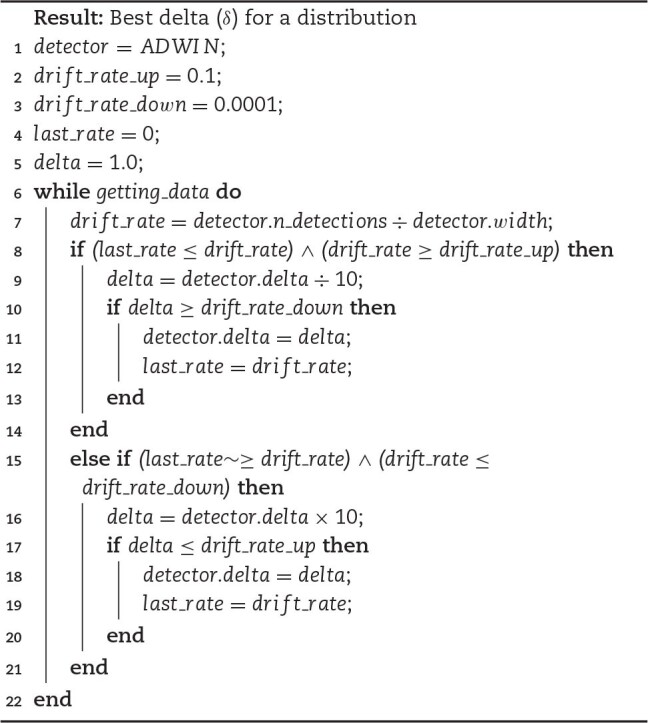
ADWIN adaptive δ for each distribution.

The δ-value in ADWIN controls how sensitive the algorithm is, and we build a training step with δ optimization for each streaming inspired by Ang et al. [53]. This training step simulates the base learners training step on ensembles. Higher δ-values admit less variation on time-series data and lower δ-values admit more variation on data, ignoring some possible drifts. Each distribution will work better with different δ, so it should be regulated.

In this article, each Analyser holds δ for 1 muscle, resulting in 8 Analysers, 1 focused on each muscle type. But if ADWIN needs to understand data and regulate δ, it has a cold start issue that alerts a drift at the beginning of the monitoring step. This problem occurs not only with ADWIN but with all CDDs that need to examine a time window to perform their analysis.

The δ is regulated using drift rate as a parameter. If the drift rate is increasing, then δ decreases, dividing it by 10. If the drift rate is decreasing, then δ increases, multiplying it by 10. There are 2 boundaries defined for high and low δ-values, and δ always starts at 1.0.

The architecture of this implementation is a simulation of ensemble with multiple base learners implemented as ADWIN and a voting system as ensemble algorithm to determine when concept drift occurs.

We show in Algorithm 1 how the ADWIN training step was implemented. The first line of the algorithm is defined CDD algorithm as ADWIN, followed by lines 2 and 3 that are initialized upper and lower boundaries for δ, respectively. Line 4 initializes the drift rate with zero indicating no changes, and line 5 assigns δ with ultrasensitive value to be regularized during the training step. During lines 6–22 δ is optimized for each streaming and "getting_data" is a function that retrieves training data; training stops when the data end. In this work the training step is built continuously for each 2 seconds of streaming data. Line 7 defines how drift rate is calculated with the accumulated number of drift detections made by ADWIN over the number of data analysed by the detector. From lines 8 to 14 the algorithm validates boundaries, and if drift rate is increasing, δ is decreased in line 11. The same happens during lines 15–21, but validating whether drift rate is decreasing, and δ is increased in line 18 if boundaries permit. "last_rate" in lines 12 and 19 is simply updated when δ changes to avoid noise.

### Experimental results

**Figure 7:**
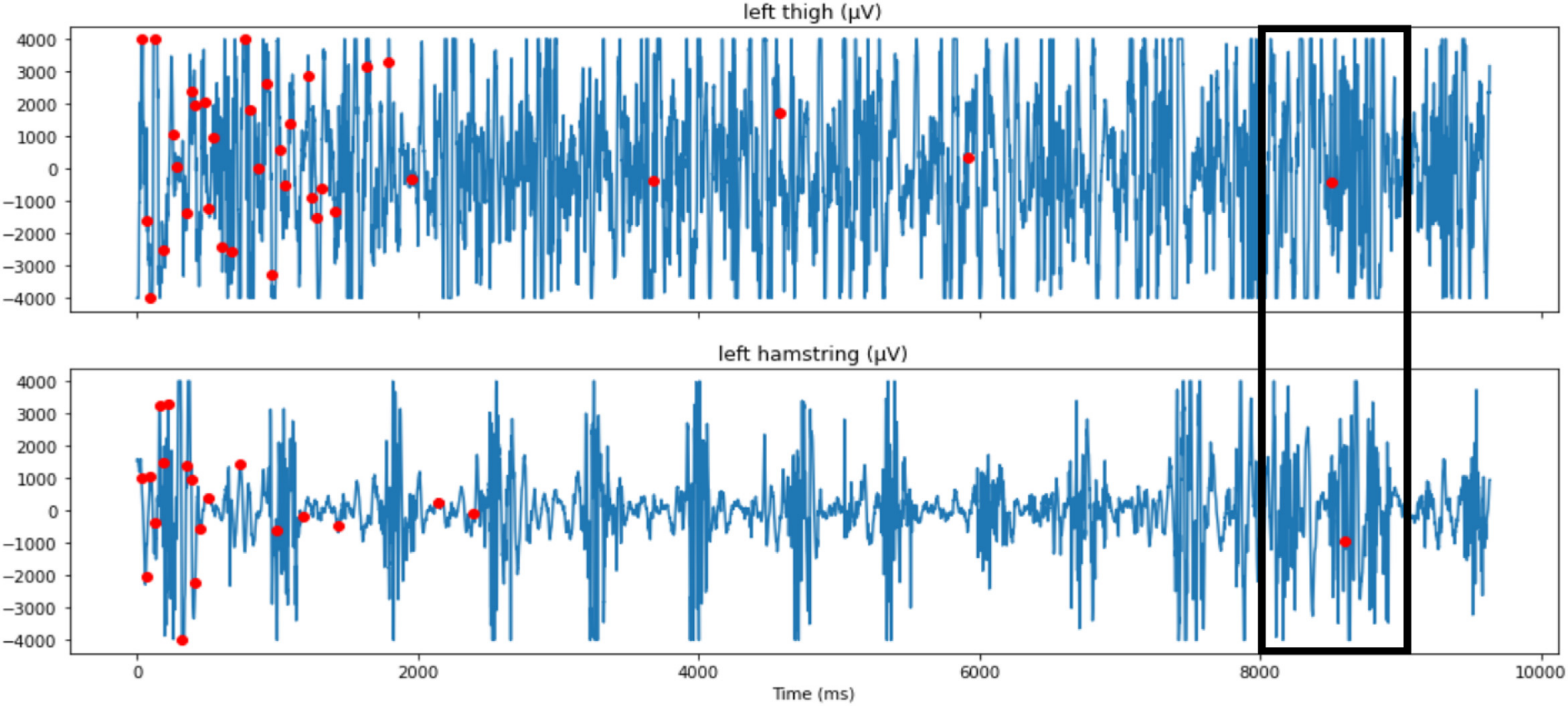
Concept drift detection of potential activity during 9,637 ms on muscles from left leg. Only 1 concept drift was detected instead of 61 by taking into account 2 muscles' signals instead of just 1.

The left leg is monitored on the results of Fig. 7, illustrating a drift detection on both hamstring and thigh muscles. It shows how even this simple Planner implementation is essential to filter some drifts at the start and during other analysis phases. Initially, no drift was detected because all muscles were adapting the δ ADWIN parameter and registering the initial distribution. The red dots indicate CDD using the ADWIN algorithm, and the black rectangle indicates when concept drift was sent to Executors because the Planner waits a 1-second time window to decide whether to fire the drift by applying its rules. So, a CDD was sent only 1 time instead of 61 times on the left leg, eliminating 60 false-positive CDDs. Starting between 7,500 and 8,000 ms, the variance of the distribution increased and ADWIN interpreted this as a drift ∼1 second later, sending an alert at 9,000 ms. It took <2 seconds to discern that probably the patient experienced muscular fatigue or moving the leg during punching exercises when this drift was detected.

**Figure 8:**
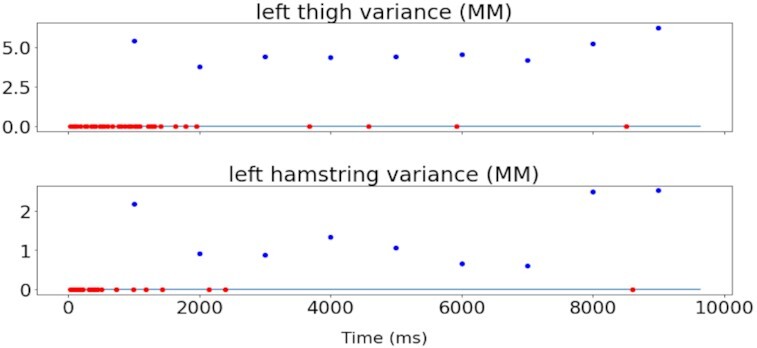
Concept drift detection related to variance. ADWIN takes variance into account when making predictions.

Using Driftage it is possible to understand its results by validating the Analysers' output and inspecting the algorithm to detect the major contributor for that result. ADWIN is very influenced by the variance of the distribution, and Fig. 8 illustrates the effect of variance on drift detection. EMG is a good application for ADWIN because the changes in variance are important for detecting changes in EMG [54]. The variance was measured for each 1-second window (blue dots), and red dots indicate detected drifts.

**Figure 9:**
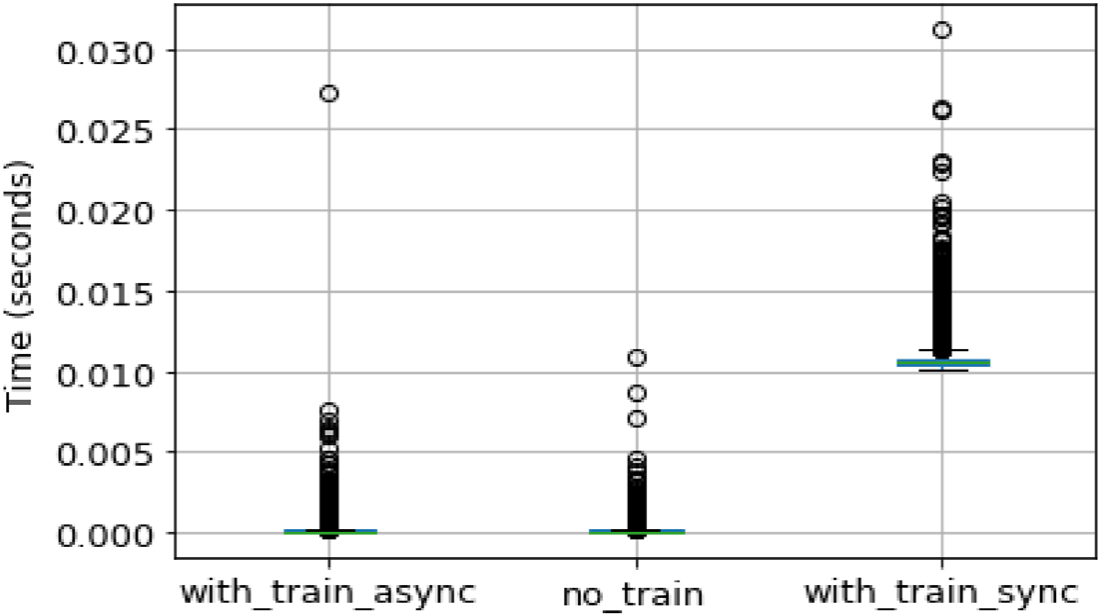
Box plot illustrating time difference when predictions are made with and without training coupled to prediction method. Green lines are the median of execution time for each experiment.

With the Driftage framework, the Analyser training phase implementation is built asynchronously by default. It is important to decouple the training phase from the prediction phase, and with Driftage it is easy to illustrate that the algorithm is scalable. Our results are illustrated in Fig. 9, where the prediction times were collected from the ADWIN Analyser with and without training phase and compared with a mimic of training and prediction code together in Jupyter Notebook: with_train_async, no_train, and with_train_sync, respectively. The median of prediction time in milliseconds for this experiment is reported in Table   2, illustrating that the training phase is not influencing prediction phase time.

**Table 2.** Median time in milliseconds for prediction with and without retraining

Status	Median time (milliseconds)
**With asynchronous training**	0.0110 (IQR: 0.0061)
**No training**	0.0109 (IQR: 0.0057)
**With synchronous training**	1.0442 (IQR: 0.0394)

## Conclusions

In this article, a multi-agent system framework, Driftage, was proposed based on MAPE-K to support CDD in distributed and scalable environments and applied to an example of health monitoring. Health monitoring was simulated by tracking the potential activity of 8 muscles but can be easily extended to other sensors. The results show the importance of concept drift in health monitoring and how 2-step validation with a Planner solves many false-positive drift detections.

All the prediction pipeline phases can be explainable and easily modularized for better predictions of concept drift or sensitivity calibration of CDDs. We can interpret the results for each step of the pipeline, as well, to measure the performance of each algorithm. One can combine ensembles of ensembles or base learners to build predictions on streams even in distributed systems because of the multi-agent system nature.

We release Driftage as an open-source framework that minimizes friction to applying new concept drift algorithms using multi-agent architecture to learn with the environment. With Driftage, data engineers and data scientists can work together with Python as a common language. Data engineers will spend most of the time with Monitors and Executors. In contrast, data scientists can build new Analysers and Planners with the researcher's custom code-building algorithms to prevent possible performance impacts during the training phase. The entire environment works well with Docker, enabling a simple adaptation and infrastructure orchestration.

This framework can be integrated with other tools for big data and streaming processing for future works, adding new Sinks and Monitors to check interoperability with more systems. Our framework works well with CDD, but we could also use it in other scenarios such as anomaly detection or online learning. Now that the framework is well defined and implemented, another future work may compare our MAS algorithms build on top of Driftage with state-of-the-art algorithms for CDD.

## Availability of Source Code and Requirements

- Project name: Driftage
- Project home page: https://github.com/dmvieira/driftage
- Operating system(s): Platform independent
- Programming language: Python
- Other requirements: Python 3.7 or higher, Ejabberd 20.04 or higher, TimescaleDB 1.7.4 or higher. Example runnable with Docker-compose 2 or higher.
- Reproducible example: https://driftage.readthedocs.io/example.html
- License: Apache License 2.0. Any restrictions to use by non-academics: No restrictions.
- Scicrunch: Driftage; RRID:SCR_021031
- BiotoolsID:   https://bio.tools/driftage

## Data Availability

Other data further supporting this work, including snapshots of our code, are openly available in the GigaScience repository, GigaDB [55].

## Editors Note

A CODECHECK certificate for this article is available confirming that Figs 7 and 8 in the article could be independently reproduced [56]. Driftage is also available as a updateable and reproducible project in Gigantum [57].

## Abbreviations

ADWIN: adaptive sliding window algorithm; API: Application Programming Interface; CDD: concept drift detection; CSV: comma-separated values; EMG: electromyography; KB: Knowledge Base; KUE: Kappa Adaptive Ensemble; MAPE-K: Monitor-Analyse-Plan-Execute over shared Knowledge; MAS: multi-agent systems.

## Competing Interests

The authors declare that they have no competing interests.

## Funding

The authors are partially supported by grants from CNPq and CAPES, Brazilian Public Funding Agencies.

## Authors' Contributions

D.M.V., C.F., and C.L. conceived the research study. D.M.V. and S.L. performed and evaluated the experiments and prepared the final version after first reviewers’ comments. All authors wrote and approved the manuscript.

## Acknowledgements

My sincere thanks to the PUC-Rio, for the support which made this work possible.

## Supplementary Material

giab030_GIGA-D-20-00288_Original_Submission

giab030_GIGA-D-20-00288_Revision_1

giab030_GIGA-D-20-00288_Revision_2

giab030_GIGA-D-20-00288_Revision_3

giab030_Response_to_Reviewer_Comments_Original_Submission

giab030_Response_to_Reviewer_Comments_Revision_1

giab030_Response_to_Reviewer_Comments_Revision_2

giab030_Reviewer_1_Report_Original_Submission Alberto Cano -- 10/26/2020 Reviewed

giab030_Reviewer_1_Report_Revision_1 Alberto Cano -- 3/8/2021 Reviewed

giab030_Reviewer_2_Report_Original_Submission Dav Joe Wada Clark, Ph.D. -- 11/2/2020 Reviewed

giab030_Reviewer_2_Report_Revision_1 Dav Joe Wada Clark, Ph.D. -- 3/22/2021 Reviewed

giab030_Reviewer_3_Report_Original_Submission Stephen J Eglen -- 12/8/2020 Reviewed
