# Supplementary material for: Driftage: a multi-agent system framework for concept drift detection
Source: Gigascience. 2021 Jun 1;10(6):giab030. doi: 10.1093/gigascience/giab030 (PMC8168350; doi:10.1093/gigascience/giab030)

GigaScience

Driftage: a multi-agent system framework for concept drift detection

--Manuscript Draft--

**Manuscript Number:**	GIGA-D-20-00288R2
**Full Title:**	Driftage: a multi-agent system framework for concept drift detection
**Article Type:**	Research
**Funding Information:**	
**Abstract:**	The amount of data and behavior changes in society happens at a swift pace in this interconnected world. Consequently, machine learning algorithms lose accuracy because they do not know these new patterns. This change in the data pattern is known as concept drift. There exist many approaches for dealing with these drifts. Usually, these methods are costly to implement as (i) they require knowledge of drift detection algorithms, (ii) software engineering strategies, and (iii) continuous maintenance concerning new drifts. This paper proposes to create a new framework using multi-agent systems to simplify the implementation of concept drift detectors considerably and divide concept drift detection responsibilities between agents, enhancing explainability of each part of drift detection. As a case study, we illustrate our strategy using a muscle activity monitor of electromyography. We show a reduction in the number of false-positive drifts detected, improving detection interpretability, and enabling concept drift detectors' interactivity with other knowledge bases.
**Corresponding Author:**	Diogo Munaro Vieira, M.D<br>PUC-Rio: Pontificia Universidade Catolica do Rio de Janeiro<br>Rio de Janeiro, Rio de Janeiro BRAZIL
**Corresponding Author Secondary Information:**	
**Corresponding Author's Institution:**	PUC-Rio: Pontificia Universidade Catolica do Rio de Janeiro
**Corresponding Author's Secondary Institution:**	
**First Author:**	Diogo Munaro Vieira, M.D
**First Author Secondary Information:**	
**Order of Authors:**	Diogo Munaro Vieira, M.D
	Chrystinne Fernandes
	Carlos Lucena
	Sérgio Lifschitz
**Order of Authors Secondary Information:**	
**Response to Reviewers:**	Editor: Please see the attached PDF with comments.<br>A "data availability" section needs to be added and the GigaDB DOI citation needs to be added and cited as well.<br>Please follow the comments in the PDF.<br><br>Authors: Done, as suggested!<br><br>Editor: In addition, please register any new software application in the bio.tools and SciCrunch.org databases to receive RRID (Research Resource Identification Initiative ID) and biotoolsID identifiers, and include these in your manuscript. This will facilitate tracking, reproducibility and re-use of your tool.<br><br>Author: Also done, and we have added as new items under "Availability of source code and requirements"<br><br>Reviewer:

Powered by Editorial Manager® and ProduXion Manager® from Aries Systems Corporation

	Two points:<br><br>1. Quite minor, the text on some of your figures is tiny. It would be nice if they were larger.<br>2. I still wish there were some "objective" validation of your EMG detection result. Maybe synthetic data. But, given the focus of the paper, it is not essential.<br><br>Authors: Thank you again for an extra careful reading and suggestions. We tried to improve the text readability on some images. All new figures are already in Github too, but unfortunately we would not have time to validade additional cases with EMG. However, it is a great opportunity for future work!
**Additional Information:**	
**Question**	**Response**
Are you submitting this manuscript to a special series or article collection?	No
**Experimental design and statistics**<br><br>Full details of the experimental design and statistical methods used should be given in the Methods section, as detailed in our Minimum Standards Reporting Checklist. Information essential to interpreting the data presented should be made available in the figure legends.<br><br>Have you included all the information requested in your manuscript?	Yes
**Resources**<br><br>A description of all resources used, including antibodies, cell lines, animals and software tools, with enough information to allow them to be uniquely identified, should be included in the Methods section. Authors are strongly encouraged to cite Research Resource Identifiers (RRIDs) for antibodies, model organisms and tools, where possible.<br><br>Have you included the information requested as detailed in our Minimum Standards Reporting Checklist?	Yes
**Availability of data and materials**<br><br>All datasets and code on which the	Yes

conclusions of the paper rely must be either included in your submission or deposited in publicly available repositories (where available and ethically appropriate), referencing such data using a unique identifier in the references and in the "Availability of Data and Materials" section of your manuscript.<br><br>Have you have met the above requirement as detailed in our Minimum Standards Reporting Checklist?	

Powered by Editorial Manager® and ProduXion Manager® from Aries Systems Corporation

Manuscript

Click here to access/download;Manuscript;Driftage_GigaScience_Journal_

OXFORD (GIGA)^n SCIENCE

GigaScience, 2021, 1–9

doi: xx.xxxx/xxxx
Manuscript in Preparation
Paper

PAPER

Driftage: a multi-agent system framework for concept drift detection

Diogo Munaro Vieira[1,*], Chrystinne Fernandes[1], Carlos Lucena[1] and Sérgio Lifschitz[1,*]

[1]Informatics Department, Pontifical Catholic University of Rio de Janeiro (PUC-Rio), Brazil

[*]dvieira@inf.puc-rio.br; sergio@inf.puc-rio.br

Abstract

The amount of data and behavior changes in society happens at a swift pace in this interconnected world. Consequently, machine learning algorithms lose accuracy because they do not know these new patterns. This change in the data pattern is known as concept drift. There exist many approaches for dealing with these drifts. Usually, these methods are costly to implement as (i) they require knowledge of drift detection algorithms, (ii) software engineering strategies, and (iii) continuous maintenance concerning new drifts. This paper proposes to create a new framework using multi-agent systems to simplify the implementation of concept drift detectors considerably and divide concept drift detection responsibilities between agents, enhancing explainability of each part of drift detection. As a case study, we illustrate our strategy using a muscle activity monitor of electromyography. We show a reduction in the number of false-positive drifts detected, improving detection interpretability, and enabling concept drift detectors' interactivity with other knowledge bases.

Key words: Concept Drift; Data Drift; Anomaly Detection; Time Series; Multi-agent Systems; Data Mining; Machine Learning Interpretability; Machine Learning Explainability

Introduction

In muscular monitoring activity, electromyography (EMG) is the primary technique to measure action potential from the muscular cells for a long time [1]. Today, several sports are using EMG, such as soccer player's athletic activity monitoring or horses searching for better equine performance [2, 3].

Machine learning techniques have been applied to EMG time series data because health monitoring needs fast insights since the patient could need emergency assistance as soon as possible [4, 5]. These data have a lot of spikes, and patterns are complicated to understand. Time series data with continuous tips are also hard to learn because all the peaks are similar to outliers or anomalies. Still, these peaks happen all the time, and we claim that algorithms need to understand that. For this purpose, concept drift strategies appear to analyze automatically streaming time series data [6, 7, 8].

There are many types of drifts in the concept drift detection (CDD) area [7, 9, 10]. Within EMG, sudden, gradual, recurring, or incremental drifts can be detected because a potential muscular activity is very reactive. When you make a move, many muscles react to it [11, 12]. There are many ways to detect each type of these drifts, and it is not elementary to build an algorithm that will detect everything. In concept drifts, there are works with supervised [13, 14], semi-supervised [15], unsupervised [16, 17, 18], statistical [19, 20], or even evolutionary algorithms [21] to deal with these drifts, but none of them is perfect for all drift types.

Some works are arising with machine learning ensembles to CDD, because of the nature of data in which these detectors should adapt with [22, 23, 24, 25]. There are several factors such as data seasonality or change of data drift type, these ensembles can choose the best estimator for each case, and each estimator can still act alone. Nevertheless, this approach needs retrain of base learners and strategies to select the best estimator that can impact detection speed [26, 22].

Compiled on: March 25, 2021.
Draft manuscript prepared by the author.

Key Points

- Multi-agent system framework to learn with environment and understand seasonal variations detecting drifts
- Framework enabling machine learning ensembles explainability and results interpretability of detected drifts
- Health sensor monitor case study using and validating the framework with real dataset
- Driftage is a modular, distributed and extensive multi-agent system framework available open source for community

One approach to designing adaptive software is using MAPE-K (Monitor-Analyze-Plan-Execute over a shared Knowledge) software pattern for self-awareness systems [27, 28, 29, 30]. MAPE-K is organized into four components:

i. **Monitor** is responsible for environmental monitoring, basically capturing data from sensors or what else the software knows about the environment and stores on Knowledge Base (KB);
ii. **Analyser** will enrich knowledge using the collected data from the environment and reporting to the KB the result of its analysis;
iii. **Planner** understands the analysis made by Analysers and assumes decisions on it while saving this information into the KB; and
iv. **Executor** gets decisions from KB and knows how to execute them. The most common representation for the Executor is an actuator.

The KB is unnecessary for all components on MAPE-K but all systems need to communicate and share information [31]. MAPE-K software architecture was recently used to model an agent on Multi-agent Systems (MAS) [32]. However, in this work, each agent is a component of MAPE-K architecture.

Traditionally, a MAS comprises agents, each of which are autonomous to learn with the environment and exchange messages with others. This nature is great to solve complex problems [33, 34]. There are some obstacles in the MAS field because of the complexity imposed when multiple smart agents communicate, particularly in distributed environments. Some of these challenges are testing the system [35] and flow consistency [36]. Moreover, streaming architectures using MAS that guide this work with best practices add less responsibility for each agent splitting intelligence between more specific agents [37, 38].

There is a lot of synergy with CDD and MAS ensembles because each agent can communicate with others and analyze the data individually to determine if the drift was detected. Some studies using MAS with ensemble strategies are already done [39, 40], but none are focused on CDD. Also, there are multiple architectures for a MAS that can be chosen to elaborate an agent-oriented software. The major challenge involves finding a good one that learns with the environment and solves the problem [41, 42, 33].

As far as the architecture for these systems enhance, the more complicated the system gets. And higher is the chance to lose control resulting in mistakes in the production environment even with some methods already described to avoid this [43, 44]. Detecting concept drifts on data streams in a scalable model for production environments is time consuming because you need to build CDD algorithms and be aware of the data pipeline, data ingestion and drift detection results [45].

To solve the dependency of data engineering pipelines and customized CDD machine learning algorithms, we propose here the Driftage [1], a modular multi-agent framework for CDD with just some types of agents that can be implemented with specialized functions deriving in other agents. Focus on a process is the best practice for MAS implementation to avoid multiple agents' complexity and improve software reuse [46, 47].

Machine learning ensembles algorithms are very complex to explain what is happening inside them and interpret its results because it is a mixture of multiple machine learning techniques and algorithms [48]. In this work, we propose a method to modularize these ensembles into various MAS agents with a much more straightforward way to explain each agent model results and even the ensemble algorithm. This MAS modularity provides both explainability for each agent's machine learning model and interpretability for its effects. Explainability and interpretability for each agent enable us to do the same for the whole system.

A case study with Driftage was created in this paper with EMG data to validate how MAS software architecture helps detect concept drifts on muscular activity during a punching exercise, enabling split efforts between scalability and drift detection issues during CDD challenges with a high extensible and modular framework.

Methods

Driftage architecture

Driftage is a modular framework based on MAPE-K, chosen as the pattern to modeling this agent-based framework because CDD needs high adaptability and fits very well with MAS.

Each agent type in Driftage has only one accountable agent on the MAPE-K architecture. Each agent can be implemented following your goal without affecting each other but can exchange information with others. Instead of an agent using the MAPE-K software pattern, an agent on the Driftage framework

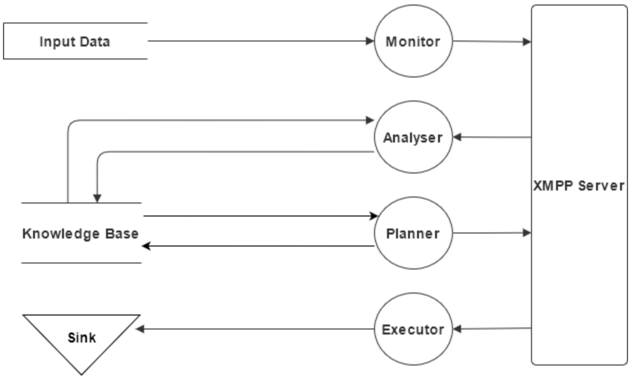

Figure 1. Driftage data flow for concept drift detection. All agents communicates through XMPP server and only the Analyser and the Planner can use information from Knowledge Base.

1 https://driftage.readthedocs.io/

Table 1. Schema of data saved in Knowledge Base.

Column	Description	Example
jid	Name of the Analyser that predicts data as drift for each collected data	custom drift analyser
data	Data collected and sent to Analyser by Monitor in JSON format	{"sensor":429}
datetime_monitored	When the data was collected by Monitor	2020-07-21 14:36:00
datetime_analysed	When the data was analysed by Analyser	2020-07-21 14:37:00
identifier	Identifier from data collected that identifies which data is monitored	left_thigh
predicted	Boolean prediction of data by Analyser representing if was detected a concept drift	false

can be implemented following one of the four types: Monitor, Analyser, Planner, or Executor. Each type can generate multiple autonomous agents.

There are 2 main flows on this framework:

i. **Monitor - Analyser:** for capture and fast prediction of concept drifts on data;
ii. **Planner - Executor:** to analyse if concept drift detected should be alerted.

These two flows can intercommunicate by a KB, where drifts are stored, and we make all history about drift analysis persistent. Each agent communicates through an XMPP server on the framework because the implementation extends Spade [2], which is a library for MAS using Python. XMPP protocol solves some problems with MAS, already providing authentication and communication channels for the agents. XMPP servers also work for load balancing and guarantee message exchanges.

We have implemented Driftage using Python because data engineers widely use it, and it enables the programmer to answer the system's requirements. The data flow for this framework is described in Fig. 1. The next section describes how we structure the KB for data sharing between flows **Monitor - Analyser** and **Planner - Executor**. These two agent communications flows are further explained in the next sections of Methods.

Knowledge Base

On MAPE-K pattern, systems are using shared knowledge that are implemented on the Driftage architecture by a database. This database stores all concept drifts that are detected and whom they were detected by, with the schema shown in Table 1.

This schema works for any relational database, and even for non-relational, which SQLAlchemy [3] supports.

Stored data collected and predicted by Analyser can be queried for retraining or by the Planner to improve your predictions. This way, we may connect the two flows. Only the Analyser and Planner know how to connect to KB.

Monitor - Analyser

This first flow is responsible for capturing data and detecting the concept drift. After drift detection, this flow saves the result on KB.

Monitor agents capture the data integrated into any framework you want: Spark, Flink, or even a Python function. Our framework send this collected data to every Analyser that asks for it. The Analyzer subscribes to Monitors to receive the data collected and analyses it using a customized predictor for CDD. Fast classifiers from Scikit-Multiflow or Facebook Prophet can be attached as a predictor.

The flow is shown as a sequence diagram in Fig. 2. After Analyser agents subscribed to Monitors, Monitors subscribe to Analysers too because they need to know if Analysers are working to send new data. When Analysers receive data from Monitors, they can predict that data and store it in the KB. There is another asynchronous task for the Analyser algorithm retraining that happens systematically.

Planner - Executor

This last flow is responsible for alerting about drifts detected. It queries KB and decides if drifts should be informed. The Planner agents keep observing for new predictions and, based on them, chooses if the drift is valid. If it is an actual drift, we should send it to the Executor. A custom predictor can be done for that too, like a voting one or a more time-consuming algorithm from Scikit-Learn, TensorFlow, PyTorch, etc. Executor agents are subscribed from Planners and receive from them new drifts to send to a custom Sink. This Sink can be an Apache Kafka, RabbitMQ, API, etc. The Executor knows if a Sink is available and informs to the Planner if it can handle new drifts. We consider a sequence diagram to describe this flow in Fig. 3.

Analyser and Planner Interaction

MAS distributed architecture make possible a lot of combinations on stream evaluation. In Fig. 4 an example of combination is using the Analysers as base learners classifying CDD on streams for further evaluation by Planner with an ensemble algorithm. Each Planner combines results from multiple Analysers that are evaluating one stream and can change Analyser subscription, ignoring it's evaluation or adding new Analysers depending of ensemble algorithm. Multiple Planners can watch same Analysers using different Executors to saves information about concept drift. It's very useful when you need to configure multiple algorithms for CDD with different sensibilities. Some machine learning models are more sensitive to data drifts than others that can wait for more aggressive data changes.

Driftage framework enables flexibility and interpretability to CDD models because each part of the CDD evaluation model can be evaluated and metrified alone. This is very useful for debugging models and understand whats happening on evaluation pipeline. For example, ensemble algorithms as Kappa Updated Ensemble (KUE) or Adaptive Random Forest[25, 24] can be implemented as Analysers combined by another ensemble algorithms on Planner. This Planner can be an ensemble algorithm or just get outside information from another KB as illustrated on Fig. 5.

Data Acquisition

There is an open dataset for electromyography on UCI Machine Learning Repository [4] with information on the activity of 8 muscles during some exercises like running, punching, or jumping. For this paper, we have chosen the punching activity file with 9.637 instances where each instance was considered as a microsecond (*ms*) action potential of a muscle cell. This

2 https://spade-mas.readthedocs.io/en/latest/
3 https://www.sqlalchemy.org/
4 https://archive.ics.uci.edu/ml/datasets/EMG+Physical+Action+Data+Set

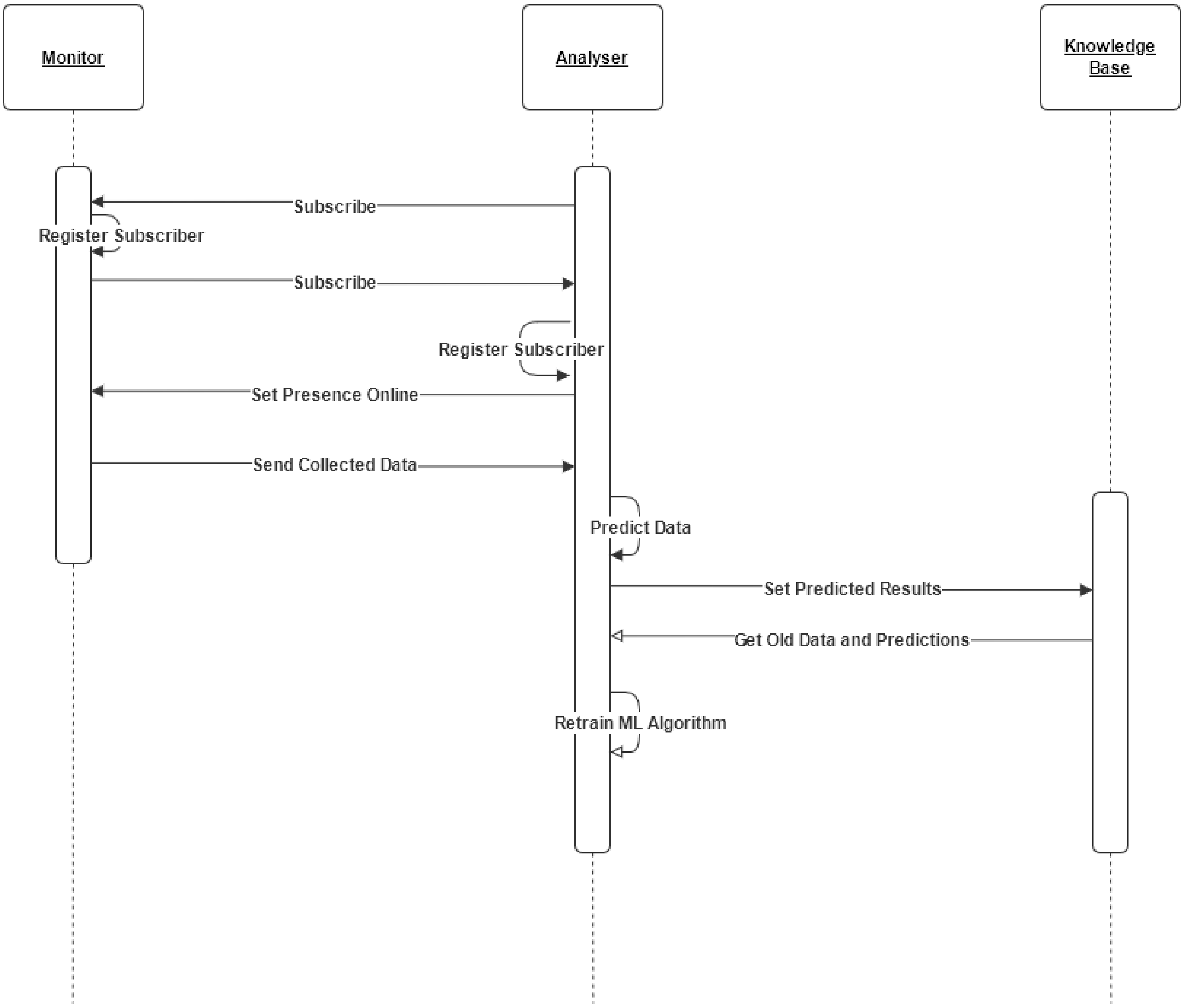

Figure 2. Monitor - Analyzer flow. The Monitor agent collects data and send to the Analyser agent only if Analyser is available, then the Analyser make predictions and saves on KB. The Analyser agent can consult KB for retraining of your predictor.

action potential is registered in microvolts (μV) at each line of the comma-separated values (CSV) file.

Health Monitor Results

This section shows the architecture proposed for a health monitor of muscle cells, following by the implementation of the CDD algorithm and the results of this algorithm on the UCI dataset to validates Driftage conception and architecture.

Architecture Design

Health monitoring is a complicated task and it's hard to know the best time to send an alert about patient health changes because there are a lot of challenges on: collect data; analyse for drifts; know the best way to communicate the drift; really send communication. The muscular activity needs more than one alteration to indicate a muscular disease or even a difference in a patient's movement. When you're doing exercises, sometimes you slightly alter your direction, but it is not characterized as muscular fatigue. An Analyser specialized for each muscle activity was developed to understand each athletic activity data and a Planner that detects if you should send a concept drift alert by Executor.

The MAPE-K based MAS framework proposed here make simple to split each responsibilities from CDD. Each agent on the architecture is responsible for: collect the data (Monitor); analyse drift on data (Analyser); decide what should do with that detected drifts (Planner) and; call the action decided as send an alert (Executor). Every agent can use the amount of Python tools to implement the best solution for each case.

Many frameworks and tools already solve monitoring and capturing data, so integrating with Apache Spark is the most straightforward approach for Python projects using PySpark. Monitors were implemented by combining with PySpark using one Monitor for each kind of muscle. For each row in a CSV file, there are eight sensor signals for each type of muscle, and Spark executors send sensor data for the corresponding Monitor. Each Monitor sends data to one Analyser but could send to others if needed. In this project design, each Analyser covers one muscle, analyzing concept drifts on it.

As long as each Analyser knows only about your muscle activity, the Planner is simple and predicts a concept drift if two or more muscles have a drift detected. We chose two muscles because the dataset has four muscles for arms and legs, two for each side (left and right), so if the patient has some problem on one side of the body, at least two muscles should be affected.

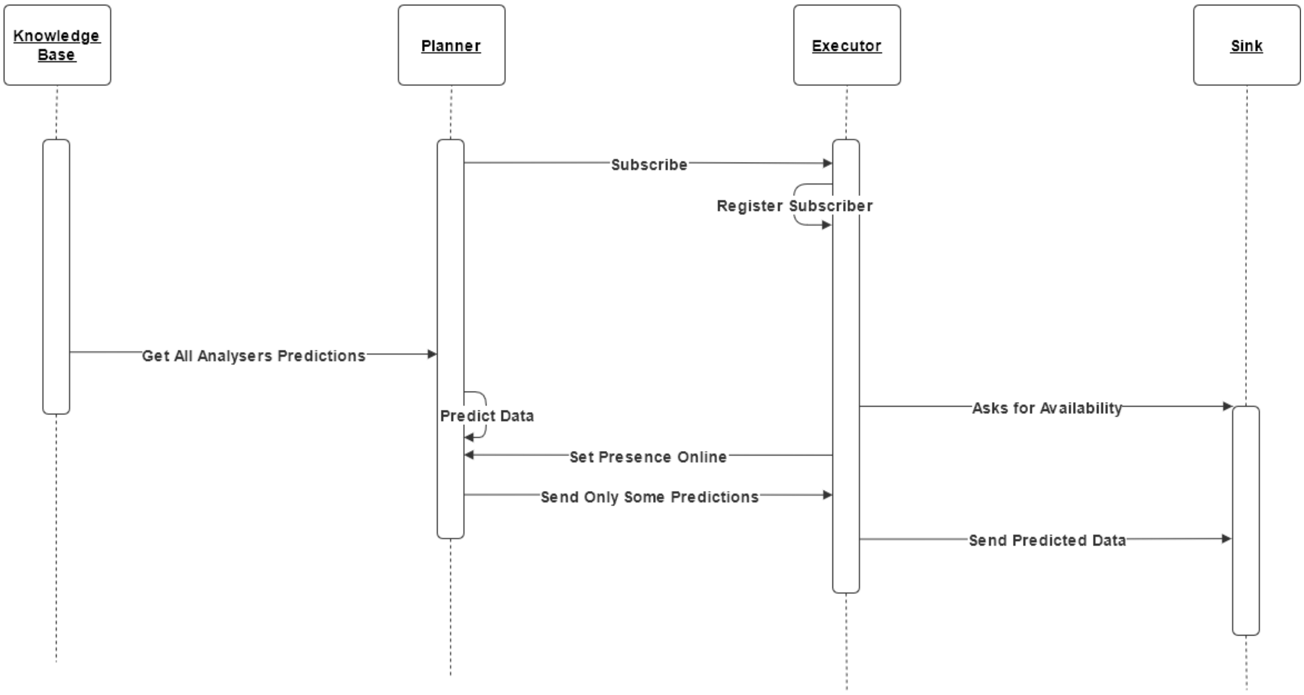

Figure 3. Planner - Executor flow. The Planner agent communicates with KB to get new predictions while the Executor agent asks Planner for concept drift detection results only if Sink is alive.

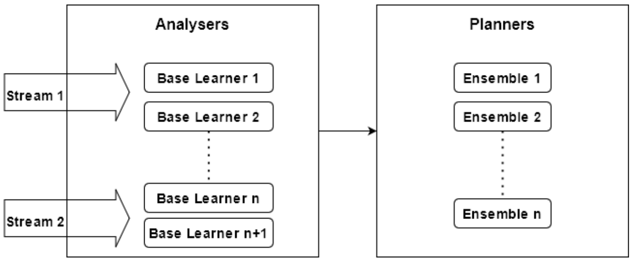

Figure 4. Driftage multi-agent ensemble abstraction and each stream been handled by two base learners.

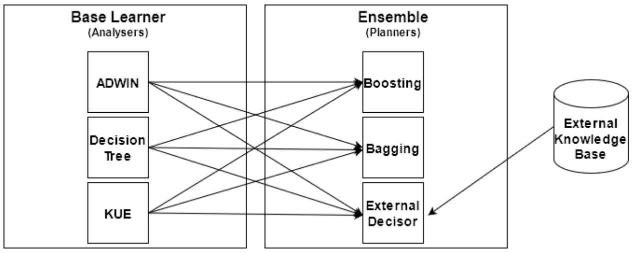

Figure 5. Every base learner and ensemble method is registered alone and your result can be interpretable without other algorithms influence.

To avoid some "cold start" problems, the Planner ignores if all the muscles indicate a drift too, so the rule is $2 \geq n\_drift < 8$ where *n_drift* is the number of muscles with drift detected. Finally, the Executor was built saving drifts on a file as CSV and validating if the file system is available to write data.

Docker containers are supporting each agent, so each Monitor, Analyser, Planner, and Executor implementations are using docker to enhance reproducibility and provides more effortless scalability. TimescaleDB as KB, uses Docker containers too during this experiment, but we recommend storing data in a better way on production environments. All this architecture design is illustrated in Fig. 6.

Drift Detection Algorithm

One of the most famous CDD algorithms is ADWIN (Adaptive sliding window algorithm) [49]. It is efficiently keeps a variable-length window of recent items; such that it holds that there has no been change in the data distribution. This window is further divided into two sub-windows (W_0, W_1) used to determine if a change has happened. ADWIN compares the average of W_0 and W_1 to confirm that they correspond to the same distribution. Concept drift is detected if the distribution equality no longer holds. Upon detecting a drift, W_0 is replaced by W_1 and a new W_1 is initialized. ADWIN uses a confidence value $\delta \in (0, 1)$ to determine if the two sub-windows correspond to the same distribution and δ is a stateless parameter in the algorithm that just contributes for CDD in the moment of compares sub-windows. ADWIN will be our base learner on ensemble architecture.

The δ value in ADWIN controls how sensitive the algorithm is and we build a training step with δ optimization for each streaming inspired by [50]. This training step simulates base learners training step on ensembles. Higher δ values admit less variation on time series data and lower δ values admit more variation on data, ignoring some possible drifts. Each distribution will work better with different δ, so it should be regulated.

In this paper, each Analyser holds δ for one muscle, resulting in eight Analysers, one focused on each muscle type. But if ADWIN needs to understand data and regulate δ, it has a "cold start" issue that alerts a drift at the beginning of the monitoring step. It is not a problem that happens just with ADWIN, but with all CDDs that need to understand a time window to analyze.

The δ is regulated using drift-rate as a parameter. If the drift-rate is increasing, then δ decreases dividing it by 10. If the drift-rate is reducing, then δ increases, multiplying it by 10. There are two boundaries defined for high and low δ values, and δ always starts at 1.0.

The architecture of this implementation is a simulation of ensemble with multiple base learners implemented as ADWIN and a voting system as ensemble algorithm to elect when con-

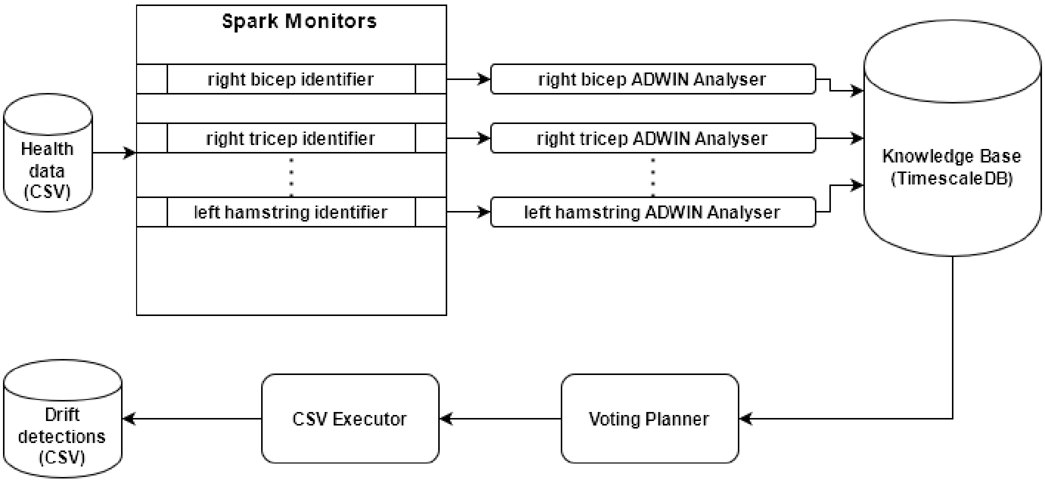

Figure 6. Health Monitor implementation. CSV file ingested by Monitors, then concept drifts are predicted by Analysers, saving it on KB. Planners get predictions and vote if is a concept drift, then the Executors save detected drifts on another CSV.

Algorithm 1: ADWIN adaptive δ for each distribution.

```
   Result: Best delta (δ) for a distribution
1  detector = ADWIN;
2  drift_rate_up = 0.1;
3  drift_rate_down = 0.0001;
4  last_rate = 0;
5  delta = 1.0;
6  while getting_data do
7      drift_rate = detector.n_detections ÷ detector.width;
8      if (last_rate ≤ drift_rate) ∧ (drift_rate ≥ drift_rate_up)
        then
9          delta = detector.delta ÷ 10;
10         if delta ≥ drift_rate_down then
11             detector.delta = delta;
12             last_rate = drift_rate;
13         end
14     end
15     else if (last_rate ≥ drift_rate) ∧ (drift_rate ≤
        drift_rate_down) then
16         delta = detector.delta × 10;
17         if delta ≤ drift_rate_up then
18             detector.delta = delta;
19             last_rate = drift_rate;
20         end
21     end
22 end
```

cept drift occurs.

We show in Algorithm 1 how ADWIN training step was implemented. The first line of the algorithm is defined CDD algorithm as ADWIN following by lines 2 and 3 that were initialized upper and lower boundaries for δ respectively. Line 4 is initializing drift-rate with zero indicating no changes and line 5 assign δ with ultra sensitive value to be regularized during training step. During lines 6-22 δ is optimized for each streaming and *getting_data* is a function that retrieves training data and training stops when data ends. In this work training step is build continually for each 2 seconds of streaming data. Line 7 defines how drift-rate is calculated with the accumulated number of drift detections made by ADWIN over the number of data analysed by the detector. From lines 8 to 14 the algorithm validates boundaries and if drift-rate is enhancing δ is decreased in line 11. The same happens during lines 15-21, but validating if drift-rate is decreasing and δ is enhanced in line 18 if boundaries permit. *last_rate* in lines 12 and 19 is just updated when δ changes to avoid noises.

Experimental Results

The left leg is monitored on the results of Fig. 7, illustrating a drift detection on both hamstring and thigh muscles. It shows how even this simple Planner implementation is essential to filter some drifts at the start and during other analysis phases. Initially, no drift was detected because all muscles were adapting δ ADWIN parameter and knowing distribution. The red dots are CDD using the ADWIN algorithm, and the black rectangle is when concept drift was sent to Executors because the Planner waits 1 second time window to fire or not the drift applying its rules. So, only one-time drift was sent instead of 61 times on the left leg, reducing 60 false positives concept drift detections. Starting between 7500ms and 8000ms, variance of distribution enhanced and ADWIN detected as drift about 1 second later, sending as an alert at 9000ms. It took less then 2 seconds to know that probably the patient was with muscular fatigue or moving the leg during punching exercises when this drift was detected.

Using Driftage is possible to understand its results validating Analysers output and inspecting inside algorithm to detect the major contributor for that result. ADWIN is very influenced by variance of distribution and in Fig. 8 is illustrated the effect of variance on drift detection. EMG is a very good case for ADWIN because the changes on variance is important to detect changes on EMG [51]. The variance was measured for each second window in blue dots and red dots are detected drifts.

Table 2. Median time in seconds for prediction with and without retraining. The same with synchronous and asynchronous training.

Status	Median Time (seconds)
with asynchronous training	0.000110
no training	0.000109
with synchronous training	0.010442

With Driftage framework, Analyser training phase implementation is build asynchronous by default. It's important to decouple training phase from prediction phase and with Driftage is easy to illustrate that the algorithm is scalable. Our results are illustrated on Fig. 9 where the prediction time were collected from the ADWIN Analyser with and without training

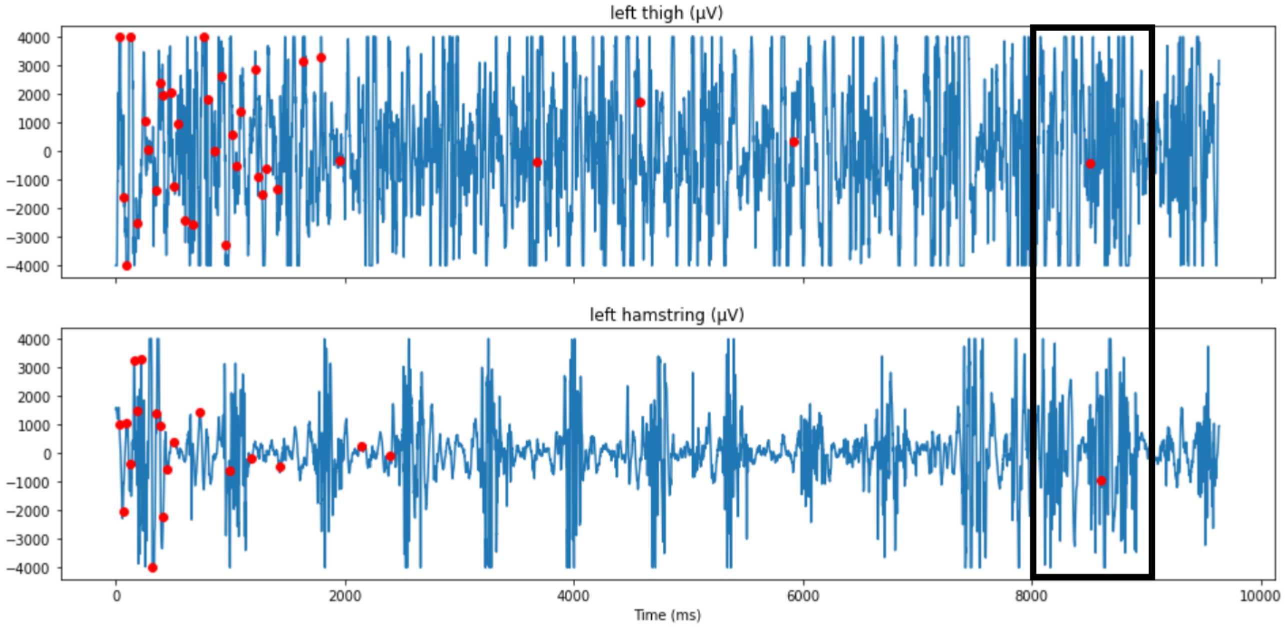

Figure 7. Concept drift detection of potential activity during 9637ms on muscles from left legs. Only one concept drift was detected instead of 61 taking in account 2 muscles signals instead of just one.

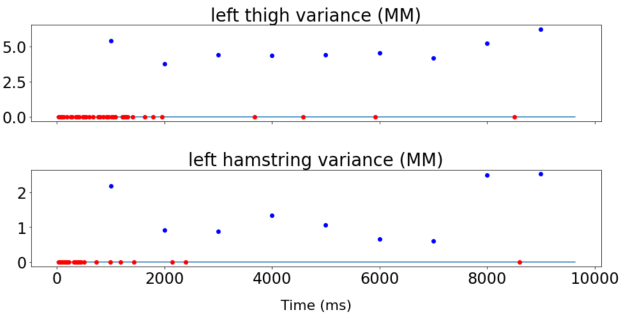

Figure 8. Concept drift detection related to variance. ADWIN take variance in account when make predictions.

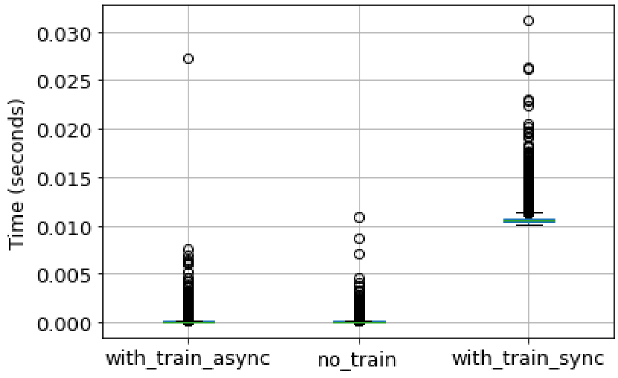

Figure 9. Boxplot illustrating time difference when you make predictions with and without training coupled on prediction method.

phase and compared with a mimic of training and prediction code together in Jupyter Notebook: *with__train__async*, *no__train* and *with__train__sync* respectively. The median of prediction time in seconds for this experiment is in Table 2 illustrating that training phase is not influencing prediction phase time.

Conclusions

In this paper, a multi-agent system framework Driftage was proposed based on MAPE-K to support CDD in distributed and scalable environments with an example of health monitoring. Health monitoring was simulated with eight muscles potential activity tracking but can be easily extended to other sensors. The results show the importance of concept drift on health monitoring and how 2-step validation with a Planner solves many false positives for drift detection.

All the prediction pipeline phases can be explainable and easily modularized for better predictions of concept drift or sensitivity calibration of CDDs. We may interpret the results for each step of the pipeline as well to measure the performance of each algorithm. One may combine ensembles of ensembles or base learners to build predictions on streams even in distributed systems because of the multi-agent systems nature.

We release Driftage as an open-source framework that minimizes friction to applying new concept drift algorithms using multi-agent architecture to learn with the environment. With Driftage, data engineers and data scientists can work together with Python as a common language. Data engineers will do most of the time with Monitors and Executors. In contrast, data scientists can build new Analysers and Planners with your custom code-building algorithms preventing possible performance impacts during the training phase. All the environment works well with Docker enabling a simple adaptation and infrastructure orchestration.

This framework can be integrated with other tools for big data and streaming processing for future works, adding new Sinks and Monitors to check interoperability with more systems. Our framework works well with CDD, but we could also use it in other scenarios like anomaly detection or online learn-

ing. Now that the framework is well defined and implemented, another future work may compare our MAS algorithms build on top of Driftage with state-of-the-art algorithms for CDD.

Availability of source code and requirements

- Project name: Driftage
- Project home page: https://github.com/dmvieira/driftage
- Operating system(s): Platform independent
- Programming language: Python
- Other requirements: Python 3.7 or higher, Ejabberd 20.04 or higher, TimescaleDB 1.7.4 or higher. Example runnable with Docker-compose 2 or higher.
- Reproducible example: https://driftage.readthedocs.io/example.html
- License: Apache License 2.0. Any restrictions to use by non-academics: no restrictions.
- RRID: *SCR*_021031
- biotoolsID : https://bio.tools/driftage

Availability of Supporting Data

Other data further supporting this work, including snapshots of our code are openly available in the GigaScience repository, GigaDB [52].

Declarations

List of abbreviations

ADWIN: Adaptive sliding window algorithm; CDD: concept drift detection; CSV: comma-separated values; EMG: electromyography; KUE: Kappa Adaptive Ensemble; KB: Knowledge Base; MAPE-K: Monitor-Analyse-Plan-Execute over shared Knowledge; MAS: Multi-agent Systems; μV: microvolts;

Competing Interests

The authors declare that they have no competing interests.

Author's Contributions

D.M.V,C.F,C.L conceived the research study. D.M.V,S.L performed and evaluated the experiments, and prepared the final version after first reviewers' comments. All authors wrote and approved the manuscript.

References

1. Moore MA, Hutton RS. Electromyographic investigation of muscle stretching techniques. Medicine and science in sports and exercise 1980;12(5):322—329.
2. Rahnama N, Lees A, Reilly T. Electromyography of selected lower-limb muscles fatigued by exercise at the intensity of soccer match-play. Journal of Electromyography and Kinesiology 2006;16(3):257–263.
3. Williams JM. Electromyography in the Horse: A Useful Technology? Journal of Equine Veterinary Science 2018;60:43 – 58.e2.
4. Guo Y, Naik GR, Huang S, Abraham A, Nguyen HT. Nonlinear multiscale Maximal Lyapunov Exponent for accurate myoelectric signal classification. Applied Soft Computing 2015;36:633–640.
5. Shi WT, Lyu ZJ, Tang ST, Chia TL, Yang CY. A bionic hand controlled by hand gesture recognition based on surface EMG signals: A preliminary study. Biocybernetics and Biomedical Engineering 2018;38(1):126–135.
6. Klinkenberg R, Rüping S. Concept Drift and the Importance of Examples. In: Text Mining – Theoretical Aspects and Applications Physica-Verlag; 2002. p. 55–77.
7. Gama J, Zliobaite I, Bifet A, Pechenizkiy M, Bouchachia A. A survey on concept drift adaptation. ACM Computing Surveys 2014;46(4):44:1–44:37.
8. Webb GI, Hyde R, Cao H, Nguyen HL, Petitjean F. Characterizing concept drift. Data Mining and Knowledge Discovery 2016;30(4):964–994. http://arxiv.org/abs/1511.03816http://dx.doi.org/10.1007/s10618-015-0448-4.
9. Lemaire V, Salperwyck C, Bondu A. A Survey on Supervised Classification on Data Streams. Lecture Notes in Business Information Processing 2015;205:88–125.
10. Lu J, Liu A, Dong F, Gu F, Gama J, Zhang G. Learning under Concept Drift: A Review. IEEE Trans Knowl Data Eng 2019;31(12):2346–2363. https://doi.org/10.1109/TKDE.2018.2876857.
11. Vezina MJ, Hubley-Kozey CL. Muscle activation in therapeutic exercises to improve trunk stability. Archives of Physical Medicine and Rehabilitation 2000;81(10):1370–1379.
12. Flint MM, Gudgell J. Electromyographic study of abdominal muscular activity during exercise. Research Quarterly of the American Association for Health, Physical Education and Recreation 1965;36(1):29–37. https://doi.org/10.1080/10671188.1965.10614654.
13. Cohen L, Avrahami G, Last M, Kandel A. Info-fuzzy algorithms for mining dynamic data streams. Applied Soft Computing 2008;8(4):1283–1294.
14. Salperwyck C, Boullé M, Lemaire V. Concept drift detection using supervised bivariate grids. In: 2015 International Joint Conference on Neural Networks (IJCNN); 2015. p. 1–9.
15. Ahmadi Z, Beigy H. Semi-supervised ensemble learning of data streams in the presence of concept drift. In: Corchado E, Snášel V, Abraham A, Woźniak M, Graña M, Cho SB, editors. Lecture Notes in Computer Science (including subseries Lecture Notes in Artificial Intelligence and Lecture Notes in Bioinformatics), vol. 7209 LNAI Berlin, Heidelberg: Springer Berlin Heidelberg; 2012. p. 526–537.
16. de Mello RF, Vaz Y, Grossi CH, Bifet A. On learning guarantees to unsupervised concept drift detection on data streams. Expert Systems with Applications 2019;117:90–102.
17. Moulton RH, Viktor HL, Japkowicz N, Gama J. Clustering in the presence of concept drift. In: Berlingerio M, Bonchi F, Gärtner T, Hurley N, Ifrim G, editors. Lecture Notes in Computer Science (including subseries Lecture Notes in Artificial Intelligence and Lecture Notes in Bioinformatics), vol. 11051 LNAI Cham: Springer International Publishing; 2019. p. 339–355.
18. Gözüaçık O, Bonab H, Büyükçakır A, Can F. Unsupervised concept drift detection with a discriminative classifier. In: International Conference on Information and Knowledge Management, Proceedings; 2019. p. 2365–2368. https://dl.acm.org/doi/10.1145/3357384.3358144.
19. L Lobo J, Del Ser J, Bilbao MN, Perfecto C, Salcedo-Sanz S. DRED: An evolutionary diversity generation method for concept drift adaptation in online learning environments. Applied Soft Computing Journal 2018;68:693–709.
20. Escovedo T, Koshiyama A, da Cruz AA, Vellasco M. DetectA: abrupt concept drift detection in non-stationary environments. Applied Soft Computing Journal 2018;62:119–133.
21. Ghomeshi H, Gaber MM, Kovalchuk Y. EACD: evolutionary

adaptation to concept drifts in data streams. Data Mining and Knowledge Discovery 2019;33(3):663–694. https://doi.org/10.1007/s10618-019-00614-6.

22. Krawczyk B, Cano A. Online ensemble learning with abstaining classifiers for drifting and noisy data streams. Applied Soft Computing Journal 2018;68:677–692.
23. Liao J, Dai B. An Ensemble Learning Approach for Concept Drift. In: 2014 International Conference on Information Science Applications (ICISA); 2014. p. 1–4.
24. Arya M, Choudhary C. Improving the Efficiency of Ensemble Classifier Adaptive Random Forest with Meta Level Learning for Real-Time Data Streams. In: Advances in Intelligent Systems and Computing, vol. 1034; 2020. p. 11–21.
25. Cano A, Krawczyk B. Kappa Updated Ensemble for drifting data stream mining. Machine Learning 2020;109(1):175–218.
26. Barros RSMd, Santos SGTdC. An overview and comprehensive comparison of ensembles for concept drift. Information Fusion 2019;52:213–244.
27. Qasim A, Kazmi SAR. MAPE-K Interfaces for Formal Modeling of Real-Time Self-Adaptive Multi-Agent Systems. IEEE Access 2016;4:4946–4958.
28. Arcaini P, Riccobene E, Scandurra P. Modeling and Analyzing MAPE-K Feedback Loops for Self-Adaptation. In: Proceedings - 10th International Symposium on Software Engineering for Adaptive and Self-Managing Systems, SEAMS 2015 Institute of Electrical and Electronics Engineers Inc.; 2015. p. 13–23.
29. de la Iglesia DG, Weyns D. MAPE-K Formal Templates to Rigorously Design Behaviors for Self-Adaptive Systems. ACM Trans Auton Adapt Syst 2015;10(3):15:1–15:31. https://doi.org/10.1145/2724719.
30. Arcaini P, Riccobene E, Scandurra P. Formal Design and Verification of Self-Adaptive Systems with Decentralized Control. ACM Trans Auton Adapt Syst 2017;11(4):25:1–25:35. https://doi.org/10.1145/3019598.
31. Petrovska A, Quijano S, Pretschner A. Knowledge Aggregation with Subjective Logic in Multi-Agent Self-Adaptive Cyber-Physical Systems. In: IEEE/ACM 15th International Symposium on Software Engineering for Adaptive and Self-Managing Systems (SEAMS '20); 2020. p. 7.
32. Qasim A, Aziz Z, Kazmi SAR, Khalid A, Fakhir I, Hassan J. Intelligent agent for formal modelling of temporal multi-agent systems. International Journal on Smart Sensing and Intelligent Systems 2020;13(1):1–13. https://doi.org/10.21307/ijssis-2020-003.
33. Seddari N, Redjimi M. Multi-agent modeling of a complex system. In: 2013 3rd International Conference on Information Technology and e-Services (ICITeS); 2013. p. 1–6.
34. Lopes Silva MA, de Souza SR, Freitas Souza MJ, de França Filho MF. Hybrid metaheuristics and multi-agent systems for solving optimization problems: A review of frameworks and a comparative analysis. Applied Soft Computing Journal 2018;71:433–459.
35. Nascimento N, Alencar P, Lucena C, Cowan D. A Metadata-Driven Approach for Testing Self-Organizing Multiagent Systems. IEEE Access 2020;8:204256–204267.
36. Zhang N, Niu W, Li T. Consistency Control of Multi - Agent System Based on Unknown Input Observer. IFAC-PapersOnLine 2018;51(31):566–571. https://www.sciencedirect.com/science/article/pii/S2405896318326028.
37. Belghache E, Georgé J, Gleizes M. Towards an Adaptive Multi-agent System for Dynamic Big Data Analytics. In: 2016 Intl IEEE Conferences on Ubiquitous Intelligence Computing, Advanced and Trusted Computing, Scalable Computing and Communications, Cloud and Big Data Computing, Internet of People, and Smart World Congress (UIC/ATC/ScalCom/CBDCom/IoP/SmartWorld); 2016. p. 753–758.
38. Twardowski B, Ryzko D. Multi-agent architecture for real-time big data processing. Proceedings - 2014 IEEE/WIC/ACM International Joint Conference on Web Intelligence and Intelligent Agent Technology - Workshops, WI-IAT 2014 2014;3:333–337.
39. Golzadeh M, Hadavandi E, Chelgani SC. A new Ensemble based multi-agent system for prediction problems: Case study of modeling coal free swelling index. Applied Soft Computing Journal 2018;64:109–125.
40. Ghosh S, Laguna S, Lim SH, Wynter L, Poonawala H. A Deep Ensemble Multi-Agent Reinforcement Learning Approach for Air Traffic Control. CoRR 2020;abs/2004.0. http://arxiv.org/abs/2004.01387.
41. Oliveira E, Pereira G, Gomes C. Reliable framework architecture for multi-agent systems interaction. In: The 7th International Conference on Computer Supported Cooperative Work in Design; 2002. p. 276–281.
42. Lakshminarayanan V, Rajashekara K, Zhu B. Multi-agent system architecture for enhanced resiliency in autonomous microgrids. IEEE Power and Energy Society General Meeting 2018;2018-Janua:1–5.
43. Li D, Ma J, Zhu H, Sun M. The consensus of multi-agent systems with uncertainties and randomly occurring nonlinearities via impulsive control. International Journal of Control, Automation and Systems 2016;14(4):1005–1011. https://doi.org/10.1007/s12555-014-0366-z.
44. Sousa COE, Custódio L. Dealing with errors in a cooperative multi-agent learning system. In: Tuyls K, Hoen PJ, Verbeeck K, Sen S, editors. Lecture Notes in Computer Science (including subseries Lecture Notes in Artificial Intelligence and Lecture Notes in Bioinformatics), vol. 3898 LNAI Berlin, Heidelberg: Springer Berlin Heidelberg; 2006. p. 139–154.
45. Gomes HM, Read J, Bifet A, Barddal JP, Gama J. Machine learning for streaming data. ACM SIGKDD Explorations Newsletter 2019;21(2):6–22.
46. Küster T, He$β$ler A, Albayrak S. Process-Oriented Modelling, Creation, and Interpretation of Multi-Agent Systems. Int J Agent-Oriented Softw Eng 2016;5(2/3):108–133. https://doi.org/10.1504/IJAOSE.2016.080892.
47. Nunes I, Kulesza U, Nunes C, Lucena CJP. A domain engineering process for developing multi-agent systems product lines. Proceedings of the International Joint Conference on Autonomous Agents and Multiagent Systems, AAMAS 2009;2:1200–1201.
48. Arrieta AB, Rodríguez ND, Ser JD, Bennetot A, Tabik S, Barbado A, et al. Explainable Artificial Intelligence (XAI): Concepts, taxonomies, opportunities and challenges toward responsible AI. Inf Fusion 2020;58:82–115. https://doi.org/10.1016/j.inffus.2019.12.012.
49. Bifet A, Gavaldà R. Learning from time-changing data with adaptive windowing. Proceedings of the 7th SIAM International Conference on Data Mining 2007;p. 443–448.
50. Ang HH, Gopalkrishnan V, Zliobaite I, Pechenizkiy M, Hoi SCH. Predictive handling of asynchronous concept drifts in distributed environments. IEEE Transactions on Knowledge and Data Engineering 2013;25(10):2343–2355.
51. Hayashi H, Furui A, Kurita Y, Tsuji T. A Variance Distribution Model of Surface EMG Signals Based on Inverse Gamma Distribution. IEEE Transactions on Biomedical Engineering 2017;64(11):2672–2681.
52. Vieira DM, Fernandes C, Lucena C, Lifschitz S, Supporting data for "Driftage: a multi-agent system framework for concept drift detection". GigaScience Database; 2021. http://gigadb.org/dataset/100882.

Table 2. Median time in seconds for prediction with and without retraining. The same with synchronous and asynchronous training.

Click here to access/download;Table;median_table.png

Table 2. Median time in seconds for prediction with and without retraining. The same with synchronous and asynchronous training.

Status	Median Time (seconds)
with asynchronous training	0.000110
no training	0.000109
with synchronous training	0.010442

Figure 1. Driftage data flow for concept drift detection. All agents communicates through XMPP server and only the Analyser and the Planner can use information from

Click here to access/download;Figure;dataflow.png

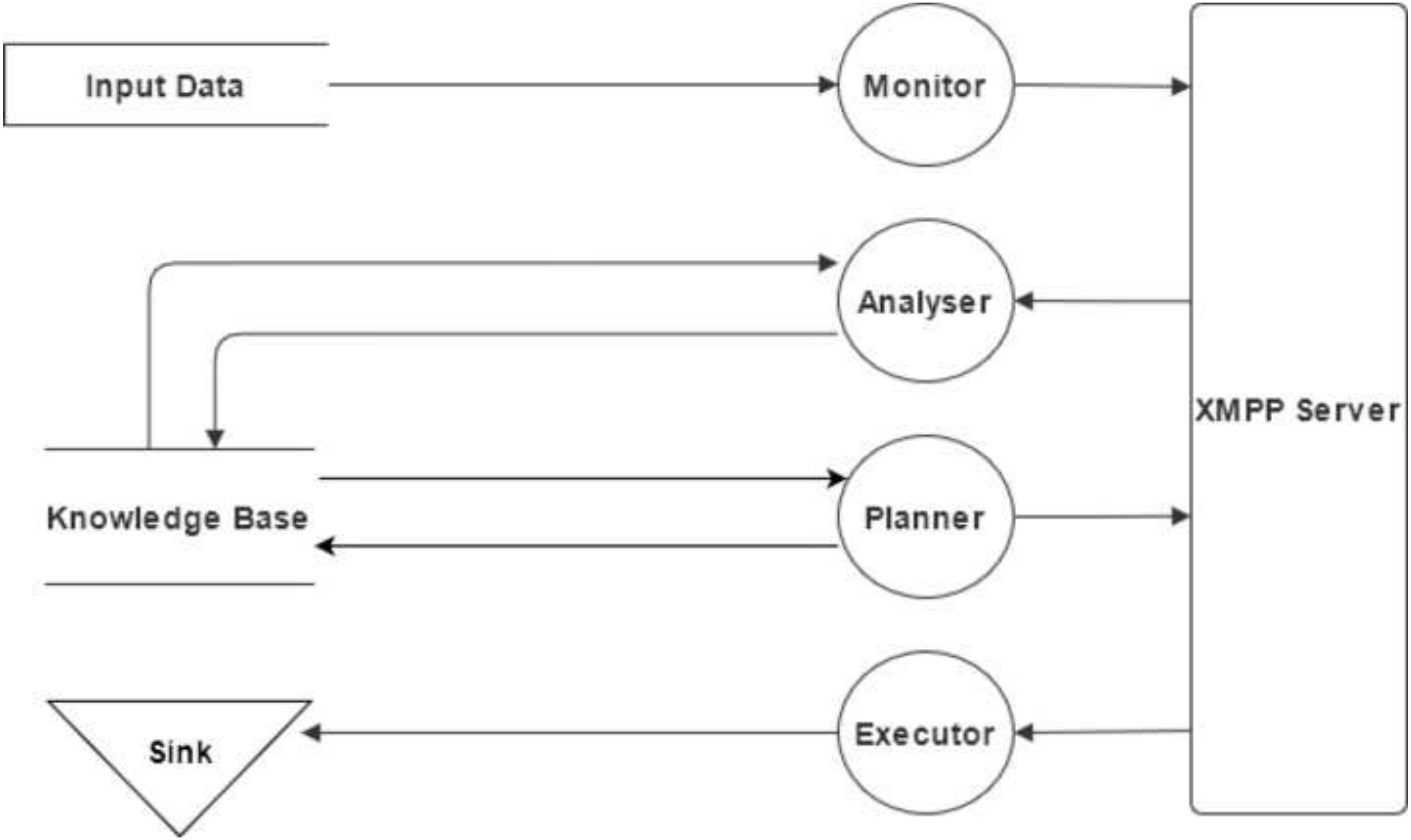

Figure 2. Monitor - Analyzer flow. The Monitor agent collects data and send to the Analyser agent only if Analyser is available, then the Analyser make predictions and

Click here to access/download;Figure;sequence_diagram_part_1.png

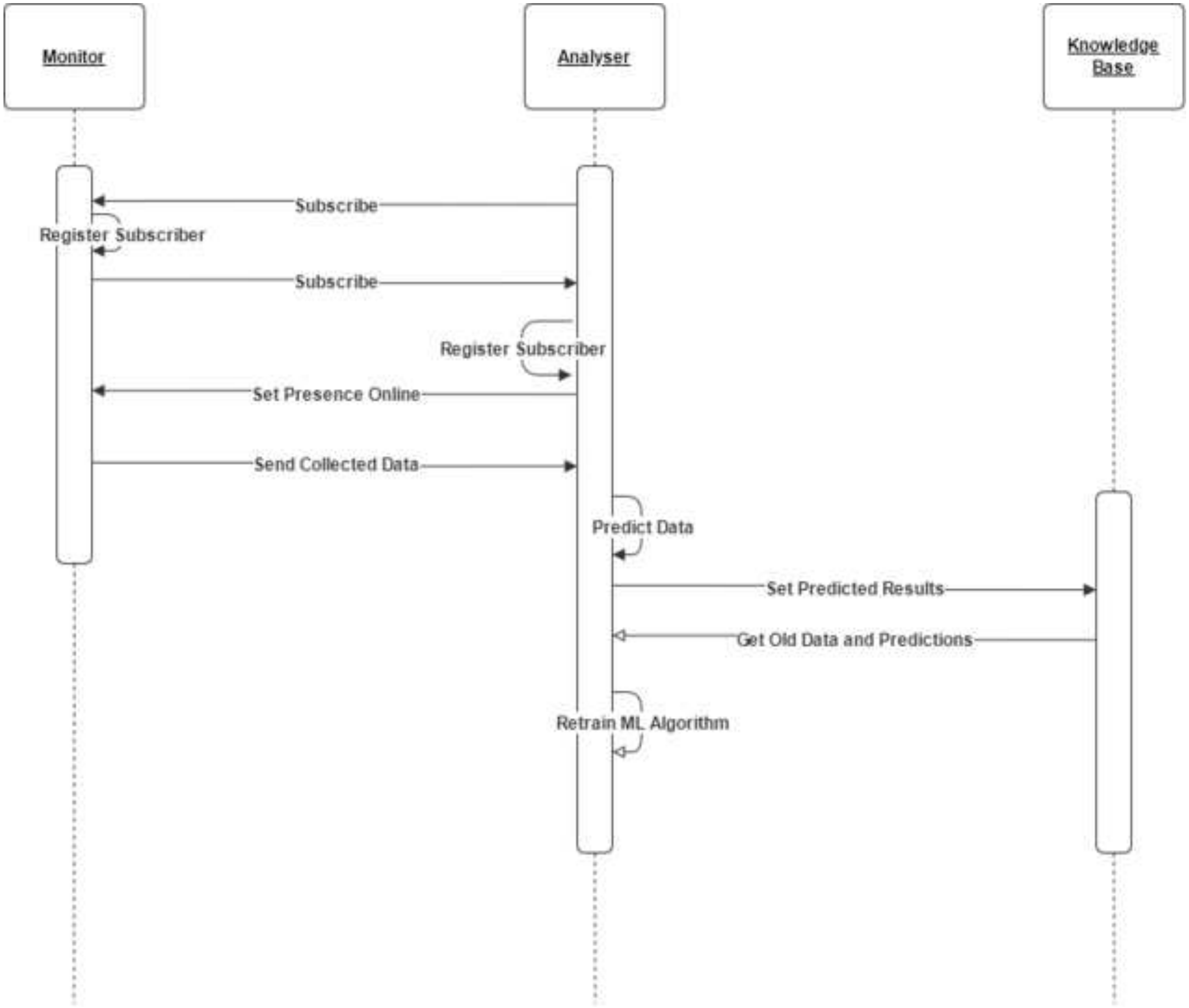

Figure 3. Planner - Executor flow. The Planner agent communicates with KB to get new predictions while the Executor agent asks Planner for concept drift detection results

Click here to access/download;Figure;sequence_diagram_part_2.png

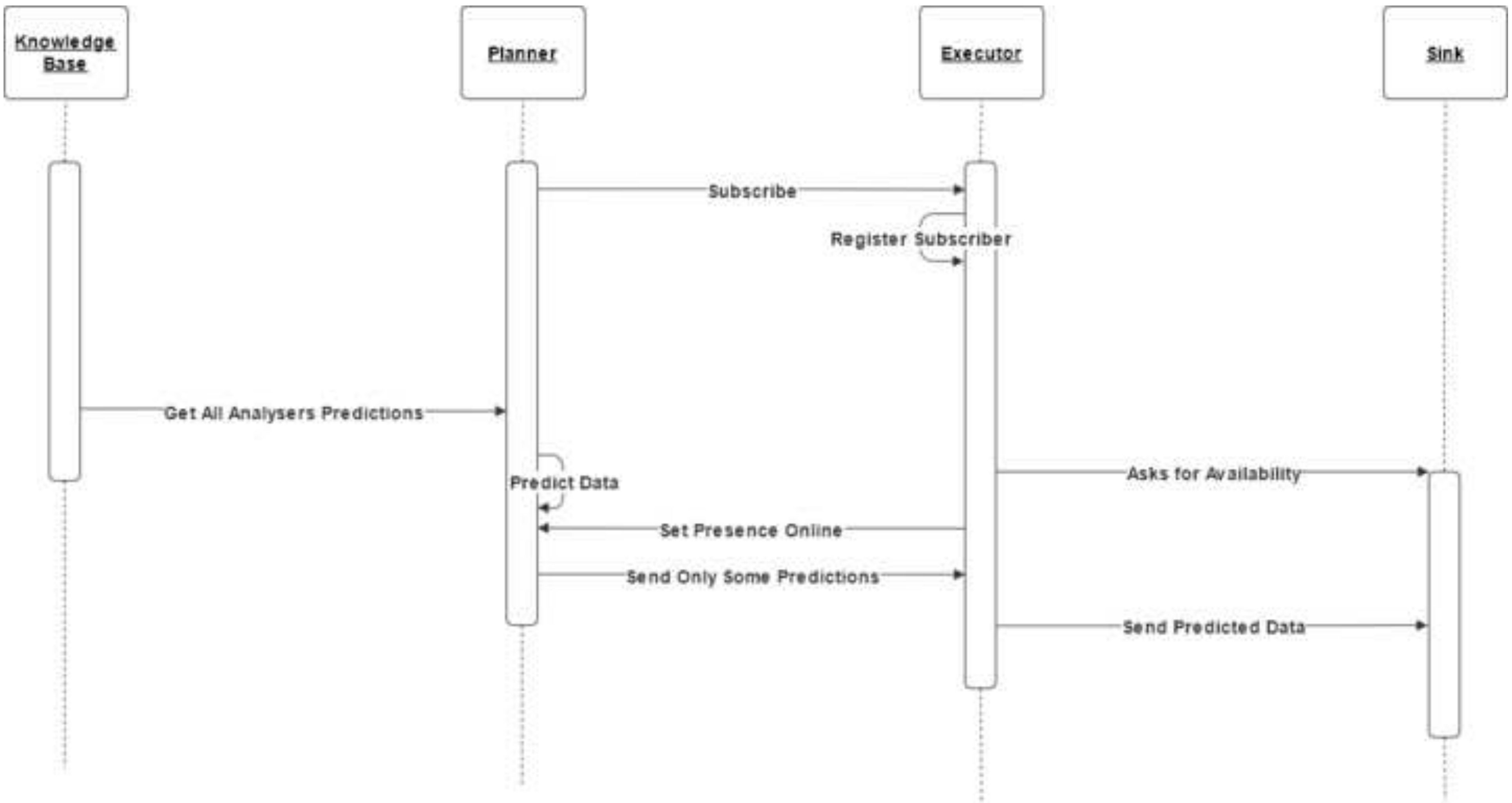

Figure 4. Driftage multi-agent ensemble abstraction and each stream been handled by two base learners. Figure

Click here to access/download;Figure;multiagent_ensemble.png

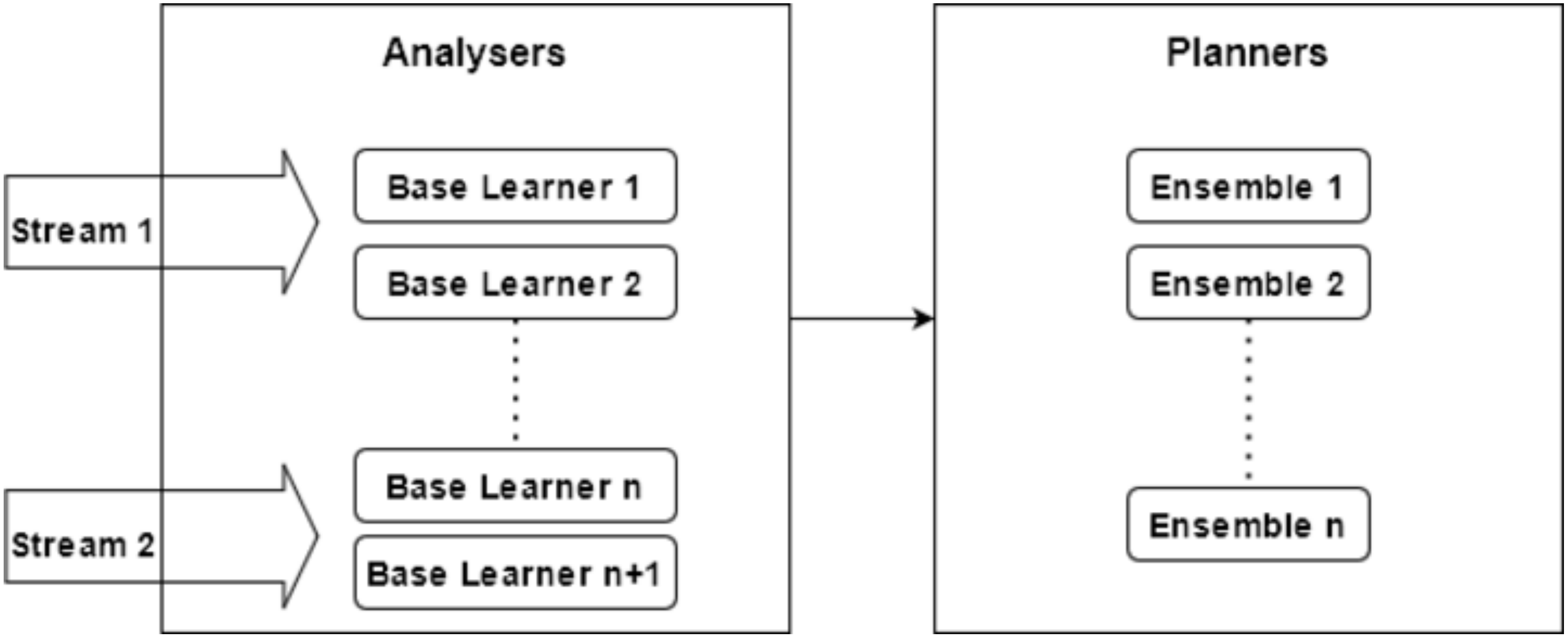

Figure 5. Every base learner and ensemble method is registered alone and your result can be interpretable without other algorithms influence.

Click here to access/download;Figure;interpretability.png

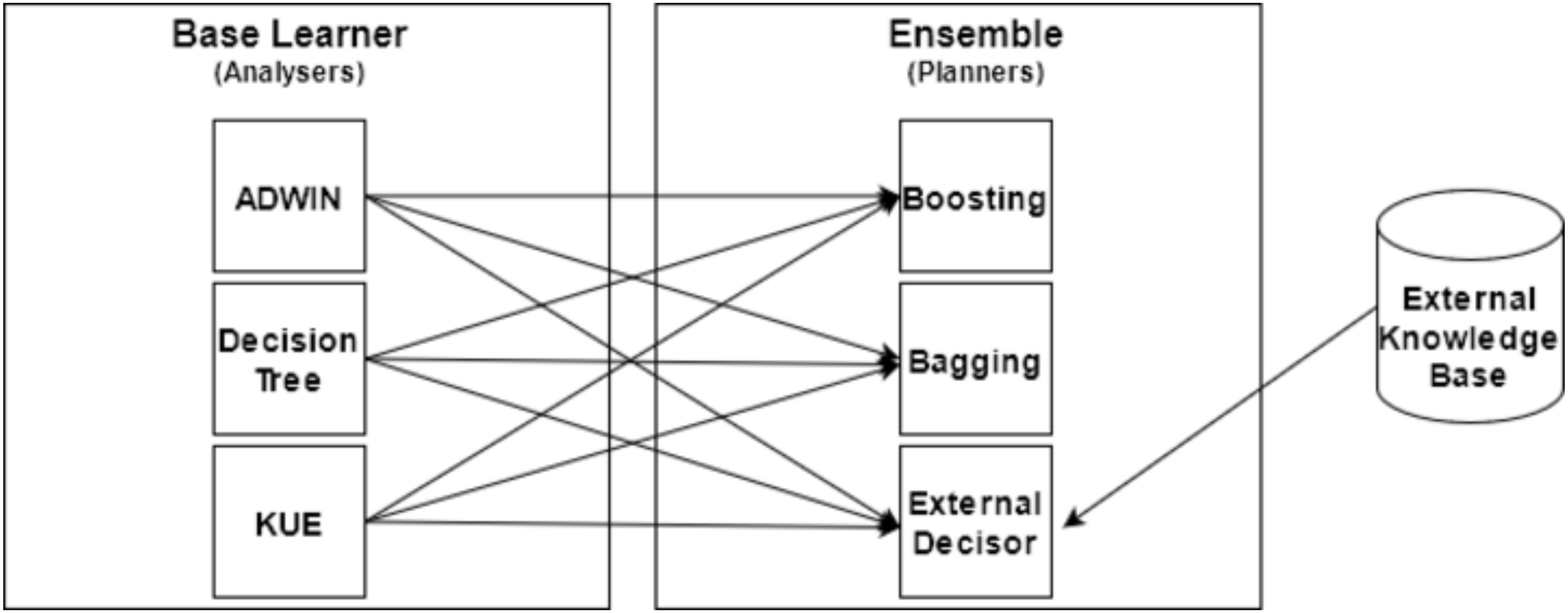

Figure 6. Health Monitor implementation. CSV file ingested by Monitors, then concept drifts are predicted by Analysers, saving it on KB. Planners get predictions and vote if

Click here to access/download;Figure;health_monitor.png

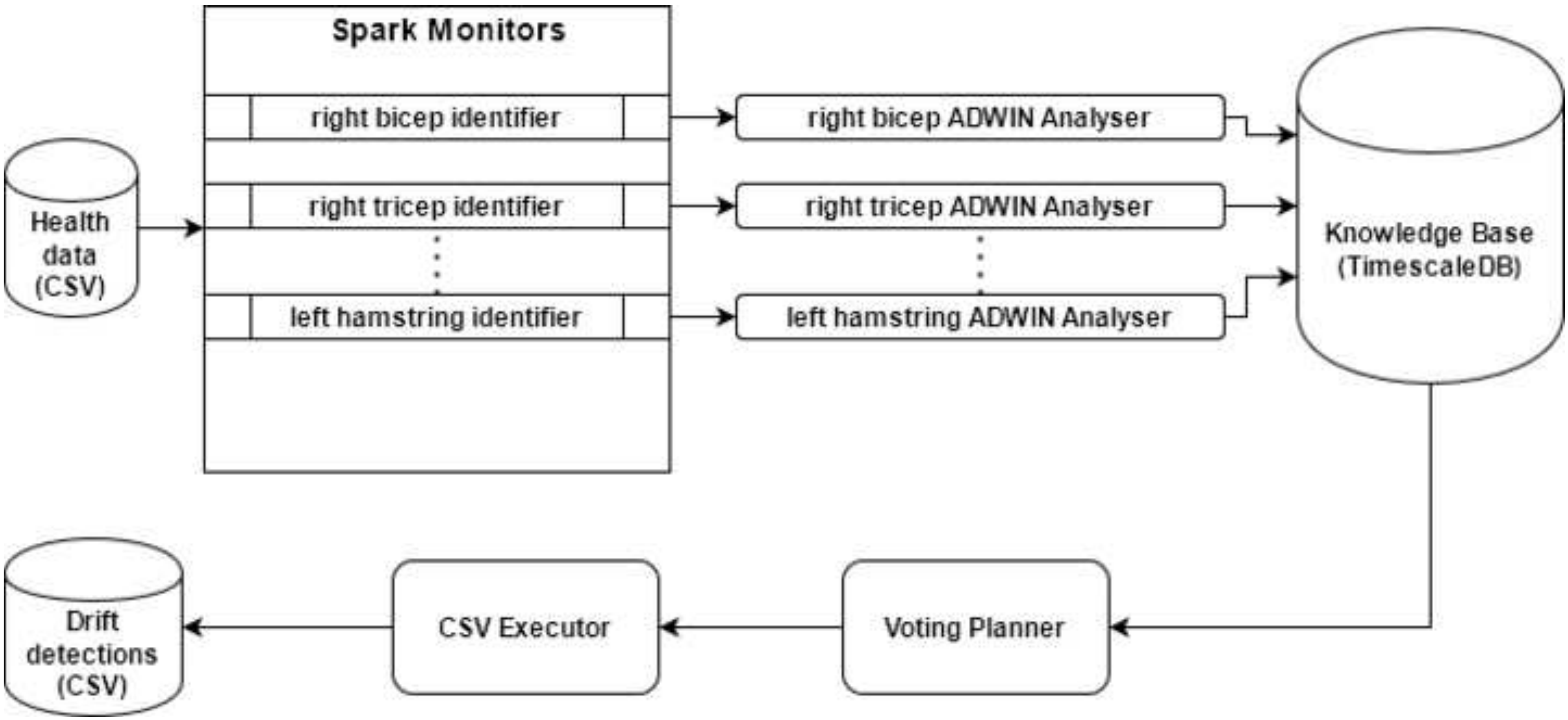

Figure 8. Concept drift detection related to variance. ADWIN take variance in account when make predictions.

Click here to access/download;Figure;variance_drift.png

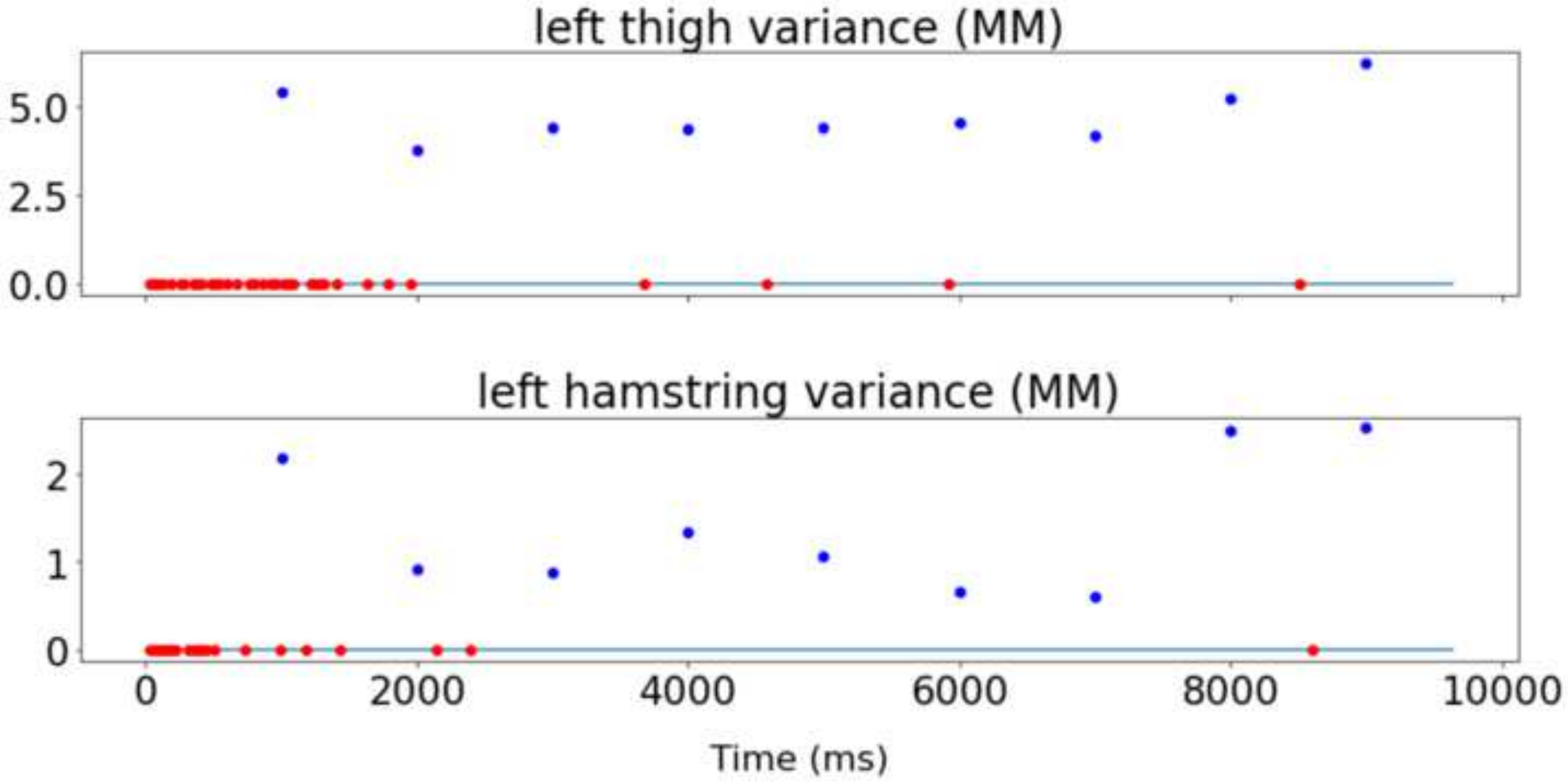

Figure 9. Boxplot illustrating time difference when you make predictions with and without training coupled on prediction method.

Click here to access/download;Figure;boxplot_time.png

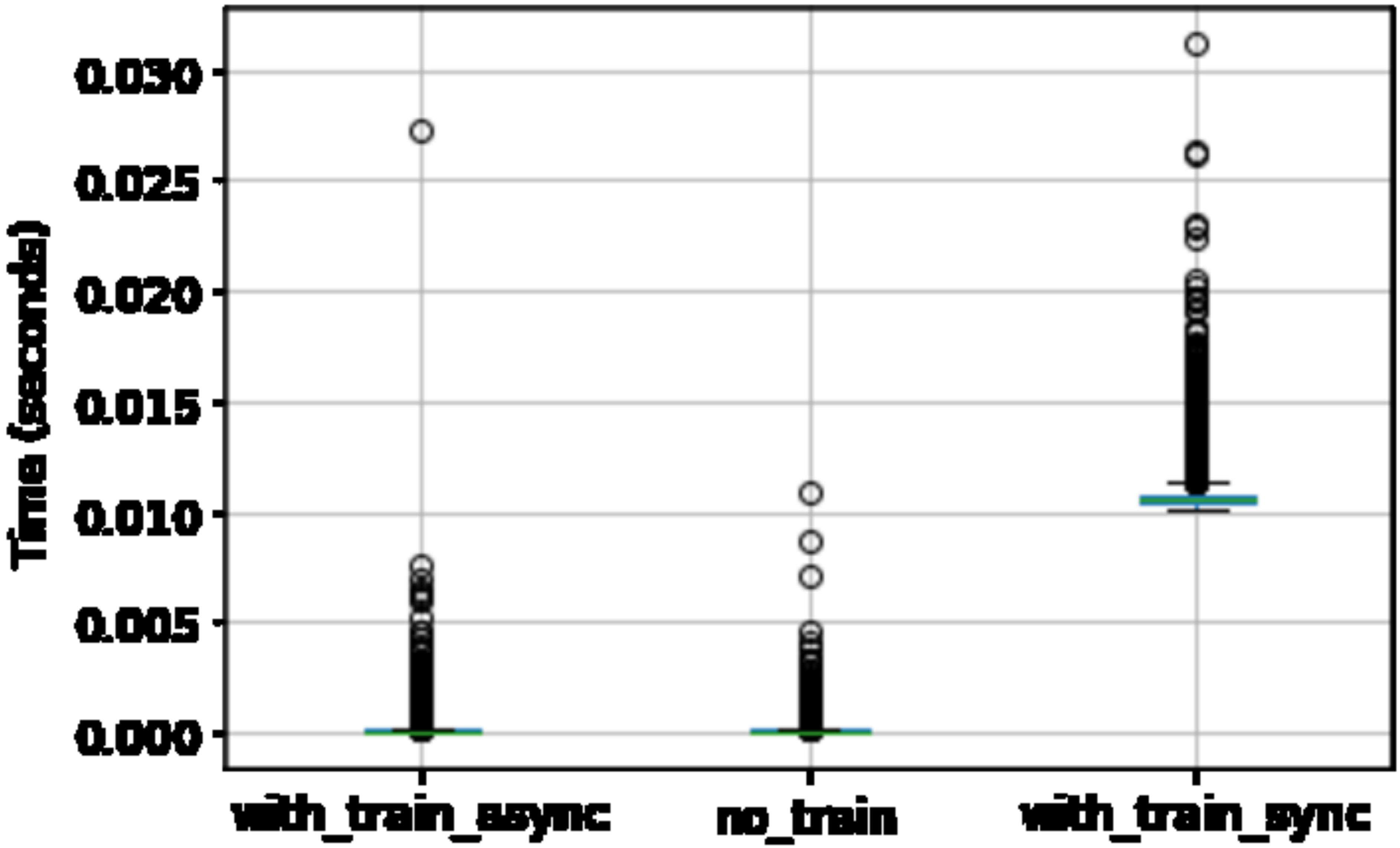

Supplement: giab030_GIGA-D-20-00288_Revision_2 [file giab030_giga-d-20-00288_revision_2.pdf]
