# Supplementary material for: Driftage: a multi-agent system framework for concept drift detection
Source: Gigascience. 2021 Jun 1;10(6):giab030. doi: 10.1093/gigascience/giab030 (PMC8168350; doi:10.1093/gigascience/giab030)
Supplement: giab030_Response_to_Reviewer_Comments_Original_Submission [file giab030_response_to_reviewer_comments_original_submission.pdf]

Author's Response To Reviewer Comments

Close

Dear Editor and Reviewers,

We thank you for your valuable comments. We have done a careful revision in the paper, considering your general and specific observations.

In the following, we describe the main changes we made to the paper and how we addressed the issues you have pointed out.

Once we have read these reviews, we realized that the work was neither describing our research work enough nor the actual benefits of using our MAS framework and that we forgot to explain a lot of points. Considering this, let us start with the main modifications we have done to improve readability and explicit our technical contributions:

- The title has changed to Driftage: a multi-agent system framework for concept drift detection to focus on the framework and not on EMG, only a case study;

- Paper highlights explain better the paper contributions;

- Added machine learning results explainability and interpretability as a benefit of the model, not clearly stated before;

- Added a new experiment to illustrate ADWIN explainability and interpretability with the variance of data and drift detection;

- Focused on the multi-agent framework benefits such as modularity, extensibility;

- Another experiment was added showing the benefits to implement ensemble methods using our framework as it modularizes the training and prediction steps;

- Examples concerning state-of-the-art algorithms that can be implemented using Driftage were included. Either ensemble algorithms can use base learners as Analysers, and ensemble method as Planners or all the ensemble can be an Analyser;

- A more detailed description about ADWIN algorithm and the delta parameter concerning self-containment;

- Enumerated ADWIN algorithm lines for the training phase and described further what each part of the algorithm was doing;

- Better explanations on detecting drifts and its relationship with ADWIN's high sensitivity to data variance;

- Added new results to Gigantum.

Reviewer 1
Reviewer: The manuscript is well written overall and has merit for publication.

Authors: Thank you! Anyway, we've worked hard to improve its readability.

Reviewer: The introduction should end with a list of the top 3-4 original scientific and technical contributions

Authors: We totally agree and we have added this list of contributions.

Reviewer: The method is well described. However, the experimental evaluation requires major extensions: both datasets/benchmarks and methods in the state of the art for concept drift (including ensembles such as Adaptive Random Forest or Kappa Updated ensemble) which adapt better to concept drift. Authors must show a complete experimental evaluation to demonstrate the superiority and generalizability of the proposed approach under a diverse set of evaluation settings compared to algorithms in the state of the art.

Authors: We really appreciate these observations. In this new version, we added two examples: KUE and ARF, and illustrated how to integrate them into the manuscript. However, we would like to point out that our primary contribution regards our software engineering architecture and not performance. This explains our focus on multi-agent systems, the modularity of ensemble algorithms, and explainability for each ensemble part. We are looking forward, in future works, to explore a performance evaluation comparison. We believe that our proposed framework may even enable a less biased evaluation.

Reviewer 2
Reviewer: The paper claims to "...create a framework using multi-agent systems to simplify the implementation of concept drift detectors." Yet, the paper dedicates significant text to exploring the example domain of EMG recordings. As such, it reads almost as two papers and the result is confusing. One topic should be clearly brought to the fore, and the impact of that topic should be clearly substantiated.

Authors: We agree; you are totally right! We have worked harder on the application but not on the framework itself and its corresponding benefits. In this new version, we enhance the framework and the expected contributions vis-a-vis the main drawbacks found in the literature. Indeed, EMG is used just as a case study to evaluate our framework in practice with a real dataset.

Reviewer: Regarding the drift detection algorithm, I've spent a decent chunk of time trying to understand what appears to be the author's modification of the algorithm, but the underlying ADWIN algorithm is not well-documented and references a SIAM paper I don't have. That said, thanks to the very clean presentation of the drift detection algorithm in Gigantum, I was able to see that the authors are modifying the delta parameter, which is documented in the underlying scikit-multiflow library as "The delta parameter for the ADWIN algorithm." Pretty circular. (1) I have no idea if it's valid to change this parameter in-flight as the authors do. Moreover, (2) I'm uncertain of the applicability of ADWIN to EMG. The label of "concept drift" implies that ADWIN was developed for something more akin to an text analysis setting. While only a few sentences could clear up this confusion, the lack of key details makes it hard to validate the use of ADWIN for EMG. Points (1) and (2) should be addressed in the text.

Authors: Once again, we agree, and the ADWIN documentation was incomplete. In this new paper version, we give more details about ADWIN, and we also explained better how our algorithm mimics a training phase of ADWIN using delta. We added some extra results about variance, too, to understand how ADWIN works concerning EMG data.

Reviewer: I have done some biological signal processing, including specifically EMG (though mostly EEG and MEG). I was expecting something like the drift problems I've seen - where the center of the signal moves. But these signals seem to stay pretty well put, with max and min values remaining similar across the timeseries for each train. I don't see meaningful drift where it's indicated with dots. On the flipside, 8 streams is not really "big data" - you can pull this off with an arduino. So the claims of applicability to big data are also unclear - I don't know if the current approach is detecting "real" drift, and it's not clear if it would scale to 100s of channels with the same kind of logic (128 channel EEGs are reasonably common, for example). Again, some clarification should be added for these points. Specifically, please guide the viewer to understand the nature of the drift if it is truly there.

Authors: Yes, it wasn't elementary to understand where the drift is when ADWIN is not clearly explained. We added a figure that illustrates how variance influences drift detection using ADWIN. Even with the same max and min values, the distribution variance changes characterizes a drift. The explanations are reflected in the manuscript. Concerning Big Data, we agree that we focus only on streaming data (velocity), and we decided to modify references accordingly.

Reviewer: While I won't demand it, I would love to see more mathematical analysis / argument / performance guarantees on the ADWIN approach. It currently feels like you just picked something that

seemed reasonable, which isn't quite computer science. BUT - there may be things that would be obvious to someone with more domain knowledge. Also, if I understand correctly, the choice of ADWIN is subsidiary to the agent-based architecture being discussed. If this is indeed a modified use of ADWIN, AND it's central to the primary claims, then I would argue more strongly for some supplementary analysis.

Authors: Thank you for these comments. We should have included in the first version a clear motivation to choose ADWIN for EMG. We confirm that for EMG analysis, it is essential to validate its variance, and ADWIN seems perfect for that. We added some text about this issue into the manuscript.

Reviewer: So, if I understand the author's intent correctly, they would benefit by greater focus on the agent-based MAPE architecture. Existing challenges that are addressed by this approach should be laid out more clearly than simply saying change detection is hard. Honestly, it doesn't *look* hard here... you even demonstrate that you can do it without your architecture in the simple example notebook in Gigantum. Perhaps another example could help round things out, or at least a description of concrete challenges that have arisen in similar situations.

Authors: These and other observations made us realize that we needed to make many improvements to our manuscript to focus on the architecture's challenges and benefits. We tried to make it more straightforward now that detecting drifts concerns collecting, analyzing data, deciding what to do with drifts, and finally executing that plan. Gigantum notebook was just a simple way to visualize results in a more controlled environment.

Reviewer: While I have been mostly critical, I applaud your efforts to produce useful open-source software with reproducible examples in docker-compose and Gigantum. I look forward to seeing a bit more explanation so that I can even better appreciate your work!

Authors: Thank you so much! Your review was incredibly detailed and pointed to many issues we have tried to solve in this new version.

Close
