# Supplementary material for: Driftage: a multi-agent system framework for concept drift detection
Source: Gigascience. 2021 Jun 1;10(6):giab030. doi: 10.1093/gigascience/giab030 (PMC8168350; doi:10.1093/gigascience/giab030)
Supplement: giab030_Response_to_Reviewer_Comments_Revision_1 [file giab030_response_to_reviewer_comments_revision_1.pdf]

Author's Response To Reviewer Comments

Close

Editor: Please see the attached PDF with comments.
A "data availability" section needs to be added and the GigaDB DOI citation needs to be added and cited as well.
Please follow the comments in the PDF.

Authors: Done, as suggested!

Editor: In addition, please register any new software application in the bio.tools and SciCrunch.org databases to receive RRID (Research Resource Identification Initiative ID) and biotoolsID identifiers, and include these in your manuscript. This will facilitate tracking, reproducibility and re-use of your tool.

Author: Also done, and we have added as new items under "Availability of source code and requirements"

Reviewer:

Two points:

1. Quite minor, the text on some of your figures is tiny. It would be nice if they were larger.
2. I still wish there were some "objective" validation of your EMG detection result. Maybe synthetic data. But, given the focus of the paper, it is not essential.

Authors: Thank you again for an extra careful reading and suggestions. We tried to improve the text readability on some images. All new figures are already in Github too, but unfortunately we would not have time to validade additional cases with EMG. However, it is a great opportunity for future work!

Close
