# Supplementary material for: Driftage: a multi-agent system framework for concept drift detection
Source: Gigascience. 2021 Jun 1;10(6):giab030. doi: 10.1093/gigascience/giab030 (PMC8168350; doi:10.1093/gigascience/giab030)
Supplement: giab030_Response_to_Reviewer_Comments_Revision_2 [file giab030_response_to_reviewer_comments_revision_2.pdf]

Author's Response To Reviewer Comments

Close

Editor:
Sorry in the last round of revisions I forgot to mark up that an "Editor's Note" section should be added and the CODECHECK certificate should also be cited for Figure 5.
Please see the attached marked up PDF for the final minor change.

Authors: It's there, but now it's Figure 7 instead of Figure 5 and Figure 8 is reproducible using the same notebook https://mybinder.org/v2/gh/dmvieira/driftage/HEAD?filepath=examples%2Fhealth_monitor%2FData%20Analysis.ipynb too. Figure 9 is reproducible using https://mybinder.org/v2/gh/dmvieira/driftage/HEAD?filepath=examples%2Fanalyse-in-memory%2FData%20Analysis.ipynb if you want to add it in CODECHECK

Close
