# Supplementary material for: Driftage: a multi-agent system framework for concept drift detection
Source: Gigascience. 2021 Jun 1;10(6):giab030. doi: 10.1093/gigascience/giab030 (PMC8168350; doi:10.1093/gigascience/giab030)
Supplement: giab030_Reviewer_2_Report_Original_Submission — Dav Joe Wada Clark, Ph.D. -- 11/2/2020 Reviewed [file giab030_reviewer_2_report_original_submission.pdf]

Reviewer Report

Title: Driftage: a multi-agent system framework for concept drift detection

Version: Original Submission **Date:** 11/2/2020

Reviewer name: Dav Joe Wada Clark, Ph.D.

Reviewer Comments to Author:

While the paper is generally well-structured and easy to follow, I feel there are critical missing pieces for the general readership of GigaScience.
The paper claims to "...create a framework using multi-agent systems to simplify the implementation of concept drift detectors." Yet, the paper dedicates significant text to exploring the example domain of EMG recordings. As such, it reads almost as two papers and the result is confusing. One topic should be clearly brought to the fore, and the impact of that topic should be clearly substantiated.
Regarding the drift detection algorithm, I've spent a decent chunk of time trying to understand what appears to be the author's modification of the algorithm, but the underlying ADWIN algorithm is not well-documented and references a SIAM paper I don't have. That said, thanks to the very clean presentation of the drift detection algorithm in Gigantum, I was able to see that the authors are modifcying the delta parameter, which is documented in the underlying scikit-multiflow library as "The delta parameter for the ADWIN algorithm." Pretty circular. (1) I have no idea if it's valid to change this parameter in-flight as the authors do. Moreover, (2) I'm uncertain of the applicability of ADWIN to EMG. The label of "concept drift" implies that ADWIN was developed for something more akin to an text analysis setting. While only a few sentences could clear up this confusion, the lack of key details makes it hard to validate the use of ADWIN for EMG. Points (1) and (2) should be addressed in the text.
I have done some biological signal processing, including specifically EMG (though mostly EEG and MEG). I was expecting something like the drift problems I've seen - where the center of the signal moves. But these signals seem to stay pretty well put, with max and min values remaining similar across the timeseries for each train. I don't see meaningful drift where it's indicated with dots. On the flipside, 8 streams is not really "big data" - you can pull this off with an arduino. So the claims of applicability to big data are also unclear - I don't know if the current approach is detecting "real" drift, and it's not clear if it would scale to 100s of channels with the same kind of logic (128 channel EEGs are reasonably common, for example). Again, some clarification should be added for these points. Specifically, please guide the viewer to understand the nature of the drift if it is truly there.
While I won't demand it, I would love to see more mathematical analysis / argument / performance guarantees on the ADWIN approach. It currently feels like you just picked something that seemed reasonable, which isn't quite computer science. BUT - there may be things that would be obvious to someone with more domain knowledge. Also, if I understand correctly, the choice of ADWIN is subsidiary to the agent-based architecture being discussed. If this is indeed a modified use of ADWIN, AND it's central to the primary claims, then I would argue more strongly for some supplementary analysis.
So, if I understand the author's intent correctly, they would benefit by greater focus on the agent-based

MAPE architecture. Existing challenges that are addressed by this approach should be laid out more clearly than simply saying change detection is hard. Honestly, it doesn't *look* hard here... you even demonstrate that you can do it without your architecture in the simple example notebook in Gigantum. Perhaps another example could help round things out, or at least a description of concrete challenges that have arisen in similar situations.
While I have been mostly critical, I applaud your efforts to produce useful open-source software with reproducible examples in docker-compose and Gigantum. I look forward to seeing a bit more explanation so that I can even better appreciate your work!

Methods

Are the methods appropriate to the aims of the study, are they well described, and are necessary controls included? Choose an item.

Conclusions

Are the conclusions adequately supported by the data shown? Choose an item.

Reporting Standards

Does the manuscript adhere to the journal's guidelines on [minimum standards of reporting?] Choose an item.

Choose an item.

Statistics

Are you able to assess all statistics in the manuscript, including the appropriateness of statistical tests used? Choose an item.

Quality of Written English

Please indicate the quality of language in the manuscript: Choose an item.

Declaration of Competing Interests

Please complete a declaration of competing interests, considering the following questions:

- Have you in the past five years received reimbursements, fees, funding, or salary from an organisation that may in any way gain or lose financially from the publication of this manuscript, either now or in the future?
- Do you hold any stocks or shares in an organisation that may in any way gain or lose financially from the publication of this manuscript, either now or in the future?
- Do you hold or are you currently applying for any patents relating to the content of the manuscript?

- Have you received reimbursements, fees, funding, or salary from an organization that holds or has applied for patents relating to the content of the manuscript?
- Do you have any other financial competing interests?
- Do you have any non-financial competing interests in relation to this paper?

If you can answer no to all of the above, write 'I declare that I have no competing interests' below. If your reply is yes to any, please give details below.

The Gigantum platform was used to illustrate the adaptive distribution change algorithm. I am a shareholder and former employee of Gigantum, Inc.

I agree to the open peer review policy of the journal. I understand that my name will be included on my report to the authors and, if the manuscript is accepted for publication, my named report including any attachments I upload will be posted on the website along with the authors' responses. I agree for my report to be made available under an Open Access Creative Commons CC-BY license (http://creativecommons.org/licenses/by/4.0/). I understand that any comments which I do not wish to be included in my named report can be included as confidential comments to the editors, which will not be published.

Choose an item.

To further support our reviewers, we have joined with Publons, where you can gain additional credit to further highlight your hard work (see: https://publons.com/journal/530/gigascience). On publication of this paper, your review will be automatically added to Publons, you can then choose whether or not to claim your Publons credit. I understand this statement.

Yes Choose an item.
